# Operative Procedures for Ultrasound Assessment of Extracranial Artery Disease: A Narrative Review by the Italian Society for Vascular Investigation (SIDV)

**DOI:** 10.3390/jcm14197050

**Published:** 2025-10-06

**Authors:** Luca Costanzo, Giacomo Failla, Leonardo Aluigi, Tiziana Anna Baroncelli, Chiara Bua, Sergio De Marchi, Elia Diaco, Federico Di Paola, Francesco Lorenzo Di Pino, Ferdinando Mannello, Ombretta Martinelli, Chiara Mascoli, Anastasia Maria Pedi, Ivan Privitera, Enrico Rescigno, Antonio Trani, Pier Luigi Antignani, Marco Mangiafico

**Affiliations:** 1 Unit of Angiology, Department of Cardio-Thoraco-Vascular, Policlinico “G. Rodolico-San Marco” University Hospital, University of Catania, 95123 Catania, Italy; jacomienko@gmail.com; 2 Unit of Angiology, Villalba Clinic, 40136 Bologna, Italy; 3 Regional Reference Center for Cancer Prevention-Villa delle Rose, 50139 Florence, Italy; t.a.baroncelli@libero.it; 4 Unit of Internal Medicine, Policlinico “G. Rodolico-San Marco” University Hospital, University of Catania, 95123 Catania, Italy; chiarabua@outlook.com (C.B.); francesco.dipino@icloud.com (F.L.D.P.); anastasia46@live.it (A.M.P.); marcomangiafico@hotmail.it (M.M.); 5 Department of Angiology, Integrated University Hospital of Verona, 37129 Verona, Italy; sergio.demarchi@univr.it; 6 Minerva Surgical Service, 88100 Catanzaro, Italy; eliadiaco@libero.it; 7 SpecialistVascular Outpatient Clinic, 95123 Catania, Italy; fedecut@hotmail.it; 8 Department of Biomolecular Sciences, University of Urbino Carlo Bo, 61029 Urbino, Italy; ferdinando.mannello@uniurb.it; 9 Vascular Surgery Division, Department of Surgery “Paride Stefanini”, Policlinico Umberto I—“La Sapienza” University of Rome, Viale del Policlinico, 00161 Rome, Italy; ombretta.martinelli@uniroma1.it; 10 Bologna Vascular Surgery Unit, IRCCS University Hospital S. Orsola, 40138 Bologna, Italy; chiara.ma@yahoo.it; 11 Department of Clinical and Experimental Medicine, University of Catania, 95123 Catania, Italy; dr.ivanprivitera@yahoo.com; 12 SIDV Board of Directors, 16100 Genova, Italy; erescigno61@icloud.com; 13 Specialist Vascular Outpatient Clinic, Provincial Health Agency, 85100 Potenza, Italy; atrani77@libero.it; 14 Vascular Center, Nuova Villa Claudia, 00161 Rome, Italy; antignanipl@gmail.com

**Keywords:** ultrasound, duplex ultrasonography, carotid disease, extracranial artery disease, vascular, carotid ultrasound, CEUS

## Abstract

**Background**: Duplex ultrasonography (DUS) of the extracranial arteries is essential for the primary and secondary prevention of ischemic stroke and the diagnosis of other cerebrovascular pathologies. The aim of the Italian Society for Vascular Investigation (SIDV) is to provide a standardised approach for the ultrasound evaluation of extracranial arterial disease. **Methods**: A multidisciplinary SIDV expert panel conducted a comprehensive literature search and performed a narrative review of the recent medical literature; the updated operative procedures were subsequently developed through a consensus-driven process that included dedicated meetings and national congress sessions for discussion and validation. **Results**: This document outlines a comprehensive approach to the ultrasound evaluation of extracranial arteries, detailing techniques such as Brightness Mode (B-mode), Colour Doppler, Power Doppler, contrast-enhanced ultrasound (CEUS), micro-vascular flow imaging (MVFI), and Three-Dimensional (3D) ultrasound. The text provides an in-depth discussion of clinical indications, technical protocols, plaque characterisation, stenosis quantification, and hemodynamic criteria. Emerging technologies are also examined for their potential to improve cerebrovascular risk stratification. **Conclusions**: The updated SIDV operative procedures for the ultrasound evaluation of extracranial cerebrovascular disease are intended to serve as a valuable reference for clinicians and vascular laboratories.

## 1. Preamble

The Italian Society for Vascular Investigation (SIDV) developed updated operative procedures for vascular diagnostic protocols, revising the previous guidelines published in 2012 [1].

A committee of experts reviewed recent medical literature on the role of ultrasound in extracranial arterial disease.

The revision process was conducted through a multidisciplinary collaboration, with contributions from specialists within the SIDV Board of Directors and external consultants. The development, drafting, and consensus-building of these operative procedures were carried out through dedicated meetings—both within small working groups and among the leadership team—supplemented by plenary sessions during the SIDV National Congress for discussion, consensus evaluation, and final approval.

The primary aim of this narrative review is to identify the most appropriate diagnostic pathways for evaluating vascular diseases and to define and standardise minimum quality requirements for non-invasive vascular diagnostics, with particular attention to evidence from the international literature.

This review encompasses the entire diagnostic process for patients with vascular disease, with a primary focus on non-invasive investigations—particularly ultrasound studies—typically conducted in diagnostic or vascular pathology laboratories. Consequently, diagnostic methods involving X-rays, magnetic resonance imaging, or nuclear medicine are not included. The guidance is not strictly compulsory for the specialist conducting the diagnostic tests. Instead, it offers evidence-based advice on diagnostic methods, their appropriateness, safety, and the efficient use of healthcare resources, always prioritising patient safety. However, following these procedures is an important ethical and professional obligation.

## 2. Introduction

Cardiovascular diseases (CVD) represent the leading cause of mortality and disability in developed countries. The World Health Organization (WHO) estimates that, in 2019, 17.9 million deaths were attributable to CVD, accounting for 32% of global deaths. Furthermore, 85% of these deaths were due to major adverse cardiac and cerebrovascular events [2]. Primary and secondary stroke prevention involves a multifaceted approach. In addition to lifestyle modifications, targeted pharmacological therapies, including antiplatelets, anticoagulants, and lipid-lowering drugs, may be required [3,4,5]. Carotid artery disease (CAD) serves as a dual indicator: it is a prominent risk factor for ischemic stroke, accounting for 10–20% of strokes or transient ischemic attacks (TIAs), and it also acts as a surrogate marker of generalised atherosclerosis and a predictor of cardiovascular events. In addition to a thorough medical history, physical examination, and laboratory assessment of metabolic abnormalities that lead to vascular changes, carotid artery imaging is a crucial diagnostic tool for evaluating a patient’s stroke and overall cardiovascular risk. The imaging of extracranial arteries represents a cornerstone of stroke prevention and plays a significant role in improving cardiovascular outcomes in other territories, such as the coronary arteries. These imaging techniques span a wide range of applications, from measuring carotid intima-media thickness (IMT) [6] to assessing plaque characteristics and their significance in terms of morphology, volume, hemodynamic repercussions [7], and even biological features in advanced disease stages [8,9,10]. Extracranial carotid steno-occlusive disease, which is typically of atherosclerotic origin, is implicated in approximately 15–20% of strokes via embolic or hemodynamic pathogenesis [11,12].

In a 2020 systematic review published in The Lancet Global Health, the prevalence of extracranial carotid atheromatous lesions was found to be 21.1% among individuals between 30 and 79 years of age, representing approximately 816 million cases worldwide [13]. The prevalence of this condition increases with age and is more common in males and white individuals [14,15]. Furthermore, atherosclerotic disease of the extracranial vertebral artery is a well-established cause of posterior circulation strokes. Proximal vertebral lesions are responsible for approximately 9% of all posterior circulation strokes. In addition, ostial lesions of the vertebral artery are implicated in another third of these cases [16,17,18]. Consequently, a detailed evaluation of extracranial vessel pathology is crucial for the appropriate risk stratification and management of patients with cerebrovascular ischemia, as well as for selected asymptomatic individuals. This assessment involves the carotid (common, internal, and external), innominate, vertebral, and subclavian arteries. The primary objective is to determine the morphological and hemodynamic status of these vessels and to identify pathologies, predominantly atherosclerotic in nature, using universally accepted criteria. Historically, the degree of luminal stenosis at the carotid bifurcation served as the primary imaging feature for determining the risk of ischemic stroke and the potential need for surgical intervention [16]. Contemporary multimodal imaging techniques, including ultrasound (US) and specific modalities such as magnetic resonance imaging (MRI), computed tomography (CT), and positron emission tomography (PET), aim to provide a more comprehensive visualisation of carotid plaque components. This enables the identification of features that indicate plaque vulnerability, such as plaque thickness and maximum volume, calcification, ulceration, intraplaque haemorrhage, lipid-rich necrotic core, and a thin or ruptured fibrous cap [19].

## 3. Materials and Methods

The primary aim of this narrative review is to provide a standardised approach for the ultrasound diagnosis and assessment of extracranial arterial disease, drawing on key insights from the medical literature. While acknowledging the inherent limitations of a narrative approach, this review is structured to provide a practical and educational resource for clinicians. A search of articles related to the topic of this paper was conducted via the use of major electronic databases, including PubMed and MEDLINE in June 2025. The search strategy utilised a combination of predefined keywords and Medical Subject Headings (MeSH) terms. Key terms included “carotid ultrasound,” “extracranial vessel disease ultrasound”, “carotid atherosclerosis”, “carotid disease”, “carotid imaging”, “carotid plaque”, “carotid stenosis”, “carotid anomalies”, “duplex ultrasound”, “contrast enhanced ultrasound”, “microvascular flow imaging”, “carotid 3D ultrasound”, “carotid endoarterectomy”, “carotid stent”, “carotid kinking”, “carotid aneurism”, “carotid web”, “vertebral artery”, “temporal artery”, “thoracic outlet syndrome”, and “subclavian artery”. There were no restrictions regarding the year of publication, and we selected only articles written in English. Articles considered relevant for the educational objective of this review were selected by a panel of experts.

## 4. Imaging Techniques for Extracranial Arterial Disease Assessment

### 4.1. Ultrasound

Carotid Doppler US represents a low-cost, widely accessible, and easily repeatable diagnostic method with an excellent safety profile. The initial duplex ultrasound (DUS) scan of a carotid artery was performed in 1972 by Eugene Strandness and Frank Barber [20,21]. The duplex principle enables the acquisition of both anatomical and physiological information about a blood vessel by integrating real-time Brightness Mode (B-mode) imaging with pulsed Doppler flow detection within a single instrument. Over time, ultrasound technology has advanced considerably, resulting in improved B-mode image resolution and alternative methods for visualising flow, such as colour Doppler and power Doppler. More recently, microbubbles have been introduced as an ultrasound contrast agent to enhance vessel assessment, thereby mitigating certain limitations like Doppler angle dependence and aliasing artefacts. These microbubble contrast agents are considered safe and carry minimal risk [22]. Given that they are not renally excreted, they can be safely administered to patients with renal failure, avoiding the risk of contrast-induced nephropathy or nephrogenic systemic fibrosis, which are known complications of other advanced imaging modalities [23].

While diagnostic ultrasound has been widely employed in clinical medicine for many years and no deleterious effects have been conclusively proven, reports of biological effects do exist. For instance, localised pulmonary capillary bleeding has been observed in mammalian systems at diagnostic exposure levels. The clinical relevance of these effects remains uncertain. Consequently, the judicious use of diagnostic ultrasound is imperative to ensure patient safety [24]. Despite a lack of comprehensive experimental data, no harmful biological effects have been demonstrated in humans, and diagnostic ultrasound has been officially declared safe. Nevertheless, physicians should remain cognizant of the potential for ultrasound-mediated biological effects, and thus, ultrasound exposure should be minimised to the level required to obtain valuable diagnostic information [25].

### 4.2. Computed Tomography

Computed Tomography Angiography (CTA) represents a relatively costly method for the simultaneous assessment of both intracranial circulation and brain parenchyma. Meta-analytic studies indicate that CTA exhibits good sensitivity (ranging from 0.68, 95% CI 0.51–0.81, to 0.77, 95% CI 0.68–0.84) and excellent specificity (ranging from 0.77, 95% CI 0.67–0.85, to 0.95, 95% CI 0.91–0.97) in the evaluation of carotid stenosis, particularly when compared to conventional angiography. Its primary advantage in acute settings is its near-universal availability in emergency departments. Furthermore, CTA can enhance the diagnostic specificity of carotid stenosis when performed after an initial ultrasound examination [26,27,28]. Carotid CTA can provide detailed information regarding the structure and composition of carotid plaques. Furthermore, CTA has demonstrated high reliability in the diagnosis of extracranial carotid occlusion and sub-occlusion. Both spiral and multi-slice CTA are considered excellent modalities for the assessment of both extracranial and intracranial vessels. Although these techniques necessitate the administration of a substantial amount of contrast, they offer crucial insights into the vessel wall.

### 4.3. Magnetic Resonance Imaging

Magnetic resonance angiography (MRA) is a relatively costly and less accessible method with several absolute contraindications. Nevertheless, it offers the significant advantage of not exposing the patient to ionizing radiation or iodinated contrast agents. Based on meta-analytic studies, MRA exhibits a sensitivity ranging from 0.76 (95% CI 0.57–0.89) to 0.88 (95% CI 0.82–0.92) and a specificity from 0.84 (95% CI 0.76–0.90) to 0.86 (95% CI 0.79–0.91) for the identification of 70–99% stenoses when compared to angiography. Similarly to CTA, MRA enables the simultaneous evaluation of brain parenchyma and intracranial circulation, often using a paramagnetic contrast agent (Gadolinium). In cases where paramagnetic contrast agents are contraindicated, information on intracranial and carotid circulation can still be acquired through specific sequences (Time-of-Flight techniques) [26,27,28,29].

### 4.4. Digital Subtraction Angiography

Digital Subtraction Angiography (DSA), which was once considered the gold standard for diagnosing extracranial and intracranial vessel pathology, has evolved to a less invasive form while maintaining and even improving its diagnostic accuracy. Consequently, it is now primarily reserved for the guidance of surgical and interventional treatments [26,27,28]. Angiography should be exclusively reserved for patients in whom a surgical or endo-vascular intervention has already been planned. A surgical team may be reluctant to plan an intervention based solely on non-invasive assessments. Therefore, angiography is also indicated when other radiological imaging is not feasible, such as in situations with a lack of appropriate equipment, when patient transport to a suitable facility is not possible, in the presence of metallic artefacts that preclude a diagnostic study, or in patients suffering from claustrophobia. Furthermore, angiography may be considered to clarify an ambiguous diagnostic pathway, to complete other investigations, or in cases of suspected vasculitis, dissections, malformations, and anomalies of the cerebral circulation [27,28].

### 4.5. Positron Emission Tomography

PET is a diagnostic modality in nuclear medicine that relies on the intravenous administration of a biologically relevant substance (e.g., glucose, methionine, or dopamine). This substance is labelled with a radioactive molecule (Fluorine 18 for glucose), and the PET scanner subsequently detects the distribution of the tracer. When clinically indicated, PET can be integrated with other radiological examinations (CT or MRI) to form hybrid systems such as PET/CT and PET/MRI. Given its ability to measure tracer accumulation, PET is valuable for identifying inflammatory diseases of extracranial vessels and for highlighting and quantifying the inflammatory activity of carotid plaques [28,30]. Consequently, PET and other diagnostic tools are indicated for the evaluation of inflammatory processes within the extracranial vasculature [26,27,28,30].

## 5. Principles of Ultrasound Imaging

### 5.1. Physic of Ultrasound

Ultrasound consists of mechanical waves that propagate through various media, such as fluids and soft tissues. These waves are generated by periodic molecular vibrations, where frequency (f), measured in hertz (Hz), is the inverse of the vibration period (T) [31]. Human hearing ranges from 20 Hz to 20 kHz; frequencies above 20 kHz are classified as ultrasound [32].

In diagnostic ultrasound, piezoelectric crystals in the probe generate sound waves from 1 to 20 MHz. These waves travel through tissues and are attenuated by reflection, absorption, and scattering. The reflected echoes are captured by the transducer and converted into electrical signals, which a computer processes to create real-time images. Grayscale B-Mode imaging uses the echo amplitude to form a 2D image. Higher-frequency waves offer better resolution but have less penetration depth due to greater attenuation, while lower-frequency waves can image deeper structures. Therefore, a balance between resolution and penetration is crucial for specific imaging needs [33].

### 5.2. Doppler Ultrasound

Colour Doppler Ultrasound Imaging (CDI) combines grayscale anatomical images with colour-coded maps of tissue velocity to visualise blood flow. It operates on the Doppler effect, where a moving object (like red blood cell) alters the frequency of reflected ultrasound waves. A higher frequency indicates movement towards the transducer (typically shown in red), while a lower frequency indicates movement away (typically shown in blue). This modality provides a qualitative, real-time visualisation of flow within a region of interest. CDI has several limitations, including angle dependence, poor resolution, and aliasing, an artefact that occurs when flow velocity exceeds the Nyquist limit [34].

Properly managing the Doppler angle is crucial in ultrasound because the angle between the ultrasound beam and the direction of blood flow determines whether a Doppler shift is detected.

- 0° angle: Blood flows toward the transducer, resulting in a strong Doppler shift.
- 180° angle: Blood flows away from the transducer, also producing a strong Doppler shift.
- 90° angle: Blood flow is perpendicular to the ultrasound beam, so no Doppler shift is detected.

This is a significant limitation, particularly when using a linear array transducer to examine vessels that run parallel to the skin surface. Since the ultrasound beam is perpendicular to the vessel, no signal would be registered. To overcome this, the beamformer electronically steers the ultrasound pulses at a 20° angle. This steering allows the Doppler sampling to be aligned with the blood flow, enabling the detection and display of Doppler signals [35].

Pulsed Wave Doppler (PWD), in contrast, uses discontinuous pulses to measure blood flow velocity within a specific sample volume. This technique produces a graphical display called a Doppler sonogram. PWD relies on parameters like the Pulse Repetition Period (PRP) and Pulse Repetition Frequency (PRF). The PRP is the time interval between two successive pulses. During this period, the probe receives echo signals from each point in the sound field to generate the B-mode image and analyse the Doppler signals. This parameter is displayed on the ultrasound machine’s monitor along with its inverse value, the pulse repetition frequency (PRF), where PRF = 1/PRP. A key artefact in PWD is also aliasing, which results from an inadequate sampling rate and can be exacerbated by a low PRF [36].

Doppler velocity measurements are more prone to errors than B-mode measurements due to several factors. A primary source of error is the beam-flow angle, as the accuracy of velocity calculations decreases with increasing this angle.

To balance signal quality and accuracy, a 60° angle is often used as a standard for velocity measurements. While angles less than 60° are more accurate, the error becomes exponentially larger at higher angles. Therefore, it’s recommended to keep the angle between 30° and 60°. Other potential sources of error include

- Difficult flow direction determination, especially in areas of narrowing (stenosis).
- Out-of-plane errors where the flow jet moves outside the imaging plane.
- Intrinsic spectral broadening and image variability can further reduce the accuracy of the measurements.

Due to these factors, errors of at least 10% are not uncommon in practice [36].

### 5.3. Power Doppler Imaging

Power Doppler imaging (PDI) is an alternative ultrasound modality that visualizes blood flow based on the amplitude of the Doppler signal rather than its velocity or direction. This technique generates a colour map that reflects the quantity of red blood cells within a given volume, creating a pseudo-angiographic effect.

The primary advantages of PDI are its angle independence and high sensitivity to low-velocity flow, making it particularly effective for visualising microvascular beds and complex pathologies like high-grade carotid stenosis [37]. PDI is clinically valuable for assessing blood flow in superficial vessels and can provide complementary information for luminal measurements in cases of high-grade carotid stenosis with complex plaque morphology [38,39]. However, PDI has some significant limitations: it does not provide information on flow direction and is highly susceptible to motion artefacts, which can degrade image quality.

### 5.4. Microvascular Flow Imaging

Microvascular Flow Imaging (MVFI) is a new ultrasound technique designed to visualise low-velocity blood flow and microvessels that are difficult to detect with traditional Doppler methods. It utilises an adaptive algorithm to minimise motion artefacts, thereby increasing its sensitivity to weak signals from small vessels. This non-invasive method improves sensitivity by suppressing flash and motion artefacts and applying adaptive filtering [40]. MVFI has shown promise in several clinical areas, including characterising lesions in organs like the liver, kidneys, and breasts [41,42,43], evaluating thyroid nodules [44], as well as evaluating vascularity in superficial organs, tendons, and nerves [45,46,47]. 

Specifically in vascular imaging, MVFI’s main applications are the assessment of intraplaque neovascularisation and post-procedural vascular complications. Studies have shown that MVFI has a firm consistency with Contrast-Enhanced Ultrasound (CEUS) in detecting intraplaque neovascularisation and endoleaks after Endovascular Aneurysm Repair (EVAR) [48,49,50]. Additionally, it has potential for assessing carotid inflammation in conditions like Takayasu arteritis [51].

Although MVFI shows promising potential, it is not yet a widely accessible ultrasound technology. Due to limited research, there are currently no established guidelines or formal recommendations for its use.

### 5.5. Contrast-Enhanced Ultrasound

CEUS has emerged as a sophisticated modality for evaluating vascular and tissue structures [52,53]. Relative to conventional ultrasound, CEUS offers enhanced detail regarding microvascularisation and tissue perfusion [54,55]. The ultrasound contrast agents consist of gas microbubbles encapsulated within lipid or protein shells [53]. Following intravenous administration, these microbubbles are confined to the vascular compartment, thereby enabling the selective visualisation of tissue perfusion [53,56].

SonoVue^®^ (Bracco Imaging S.p.A., Milan, Italy) is the most commonly used contrast agent for vascular applications [52]. Its composition includes sulphur hexafluoride microspheres encapsulated within a phospholipid monolayer [53]. The microbubbles possess a small average diameter of 2.5 μm, which confines them to the vascular compartment and prevents them from extravasating across the endothelium [53]. In contrast to CT and MRI contrast agents, ultrasound contrast microbubbles are directly altered by the acoustic waves used for their detection [56]. The application of low acoustic energy (mechanical index ≤0.1) induces stable oscillation of the microbubbles without causing their destruction. This process generates harmonic signals, which are subsequently detected by specially configured ultrasound equipment [53,56], thereby facilitating highly sensitive imaging of tissue microvascularisation.

#### 5.5.1. Application of CEUS

In recent years, the clinical utility of CEUS has expanded significantly across various fields. It is widely applied in the assessment of focal liver lesions [57], the examination of renal pathologies [58], cardiological investigations [59], vascular assessment [60], and in the evaluation of pancreatic pathologies [61]. Furthermore, CEUS has proven valuable in the diagnosis of inflammatory and infectious diseases [62].

Within vascular imaging, CEUS has demonstrated utility in assessing atherosclerosis of the supra-aortic trunks, with a specific focus on the carotid arteries [63].

CEUS is more reliable than conventional Doppler techniques for the detection of slow blood flow and severe stenosis [64]. It provides better visualization of blood flow and more accurate delineation of the vessel wall, particularly in cases of severe carotid stenosis or carotid near-occlusion [65,66], allowing for differentiation of these conditions, thereby guiding appropriate patient management [67].

In addition to stenosis evaluation, CEUS facilitates the assessment of carotid plaque morphology, specifically ulceration and intraplaque neovascularisation [68,69].

These morphological characteristics represent crucial criteria for the identification of vulnerable atherosclerotic plaques. Intraplaque neovascularisation, as detected and quantified by CEUS, serves as a significant marker of plaque vulnerability [70]. The presence of fragile, permeable capillaries within the plaque indicates a higher degree of vulnerability [71]. CEUS is effective at detecting and quantifying this neovascularisation because the contrast agent microbubbles behave and are sized similarly to red blood cells, which allows them to reflect the plaque’s microvascularisation [72]. Several studies have shown a strong correlation between CEUS contrast enhancement and the histological vascular density of carotid plaques [73,74,75,76].

Furthermore, CEUS can be a valuable tool in the diagnosis of carotid dissection, as it enhances diagnostic accuracy. This is particularly relevant for distinguishing the true from the false lumen, a task that often proves challenging with conventional ultrasound [64].

CEUS has emerged as an effective diagnostic modality for large vessel vasculitis, including giant cell arteritis (GCA) and TAK. In a pilot study on GCA, CEUS demonstrated the capability to visualise increased perfusion within inflamed arterial walls, which offers a potential advantage over standard ultrasound, a technique that primarily identifies the “halo sign” [77]. For TAK, Ma and coworkers observed enhanced arterial wall perfusion, which was evaluated with CEUS during the active disease phases [78]. These initial findings were subsequently corroborated by two additional studies [79,80].

A principal advantage of CEUS in the context of vasculitis is its capacity to provide a more direct assessment of disease activity compared to standard ultrasound, which predominantly relies on morphological changes. Nevertheless, due to its associated cost and the logistical complexity related to contrast agent administration and venous access, the clinical application of CEUS remains largely confined to research settings [81].

#### 5.5.2. CEUS Protocol [82]

Ultrasound Equipment Preparation

- Use an ultrasound machine with contrast-specific imaging modes (e.g., pulse inversion).
- Set the mechanical index to a low value (≤0.2) to keep the microbubbles intact.
- Use of a linear probe with a frequency range of 3–11 MHz

Contrast Agent Administration

- Inject a 2.4 mL bolus of SonoVue^®^ (Bracco Imaging S.p.A., Milan, Italy), a sulfur hexafluoride-based contrast agent, into a large vein, like the antecubital vein, to prevent microbubble damage.
- Follow the injection with 5–10 mL of 0.9% saline to ensure the contrast circulates properly.

Image Acquisition

- The contrast agent typically appears in the carotid arteries within 20–30 s after injection.
- Record a cine loop lasting 280–360 s.

Image Analysis

- Perform a qualitative assessment of the recorded cine loops.
- Conduct a post-processing analysis to measure parameters like intraplaque enhancement intensity.

### 5.6. 3D

3D US has been established over the past 30 years as a valid and reproducible method for characterising plaque morphology [83]. Multiple techniques are available for acquiring 3D ultrasound images. Initially, techniques were developed using a two-dimensional (2D) linear array transducer with mounted positional sensors such as optical or electromagnetic field tracers. These systems require additional equipment and careful calibration. This technique is not error-free due to possible electromagnetic interference artefacts and is inherently operator-dependent, as errors can arise from movements during scanning, manual tracking, and post-procedural analysis [84].

More recently, a 3D phased-array scan was introduced. This technique employs a specialised transducer incorporating 2D crystal arrays and dedicated software to generate 3D images in real time. Unlike previous methods, transducer movement is not required, as the phased arrays electronically sweep the target volume to create volumetric images [85].

The main advantages of carotid 3D US include the ability to measure a specific lesion in all planes, which allows for longitudinal disease monitoring, and the identification and quantification of plaque ulcers. Further research on plaque vulnerability characteristics is necessary to stratify risk and to establish threshold cutoff values that can optimally predict CVD risk [86]. The current use of 3D echo in carotid disease diagnostics has several limitations, including the complexity of acquisition, heterogeneity of protocols, and a lack of standardisation. Therefore, currently, 3D is more of a support and research tool than a routine method.

## 6. Carotid Artery Disease

### 6.1. Indications for Ultrasound Scan

The primary indications for ultrasound scans include symptoms of TIA and acute cerebrovascular events (stroke). According to the latest guidelines, carotid imaging (using US, CTA, or MRA) should be performed within 24 h in patients with ischemic stroke or TIA who are deemed candidates for carotid intervention after specialist [87] assessment [87]. Other key indications are

- Transient monocular blindness (amaurosis fugax);
- Follow-up of known carotid stenosis;
- Preoperative evaluation before major surgical procedures, especially cardiothoracic;
- Post-intervention follow-up (carotid endarterectomy, stent, etc.);
- Suspected subclavian steal syndrome;
- Pulsatile neck mass;
- Rheumatic disease with vascular involvement.

Ultrasound examination of the supra-aortic trunks as a screening tool is not recommended in asymptomatic patients without significant risk factors. Furthermore, dedicated guidelines do not recommend the use of carotid Doppler ultrasonography in patients presenting with typical syncope [88]. However, US screening may be indicated for

- Asymptomatic individuals at high risk, such as patients with peripheral artery disease, severe coronary artery disease, on haemodialysis, with a carotid bruit, with previous neck irradiation, or those aged over 60 years with at least two cardiovascular risk factors [19,89,90].
- Asymptomatic individuals with elevated blood pressure or hypertension, specifically when the results are likely to alter patient management [91]

According to the European Society of Cardiology (ESC) guidelines, the presence of carotid plaque, as assessed by ultrasound, may be considered a risk modifier in patients at low to moderate cardiovascular risk, as well as in those with hyperlipidemia and diabetes [92,93,94]. These guidelines also do not recommend IMT for CVD risk assessment.

### 6.2. Equipment

Proper transducer selection is essential for carotid artery duplex evaluation. When choosing a transducer, it’s critical to consider the transmitted frequency for optimal image quality and the transducer footprint for access and visualisation. A higher frequency transducer is typically required for superficial structures, whereas a lower frequency transducer is necessary to adequately visualise deeper structures. Typically, linear probes with a frequency range of 5 to 12 MHz are used, with lower frequencies employed for PWD and colour Doppler functions. The examination involves real-time two-dimensional visualisation of anatomical structures, the use of colour modules, and spectral analysis. The acquired data are then thoroughly documented. Microconvex probes are also used to evaluate more challenging segments or in cases of complex neck anatomy. Hockey stick probes are small-footprint linear array transducers with high frequency (18–22 MHz) that are utilised to assess small superficial structures, such as the temporal arteries.

### 6.3. Methodology of Examination

**Figure 1:**
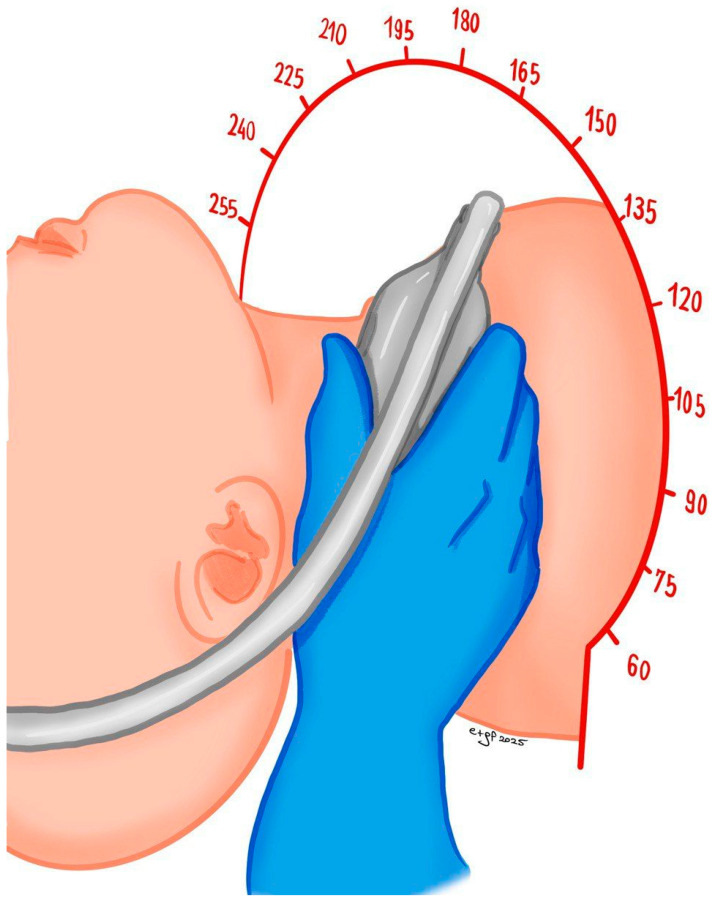
Patient and operator position for carotid ultrasound study.

The patient should be positioned supine with their head and shoulders resting on a pillow, and their head should be turned slightly away from the side being examined. We recommend that the operator sit behind the patient, with their scanning arm resting on the patient’s bed and the opposite arm operating the ultrasound system (Figure 1).

Alternatively, the operator may also sit beneath the table and perform the examination from an anterior approach.

The examination starts from the base of the neck, positioning the transducer in a transverse plane to explore the common carotid artery (CCA), the proximal CCA, the brachiocephalic artery (on the right), the subclavian artery, and the origin of the vertebral artery. Subsequently, the scan proceeds cranially along the CCA to the carotid bifurcation, moving distally from the carotid bifurcation to evaluate the internal carotid artery (ICA) and the external carotid artery (ECA), documenting any pathology present. Finally, the transducer is oriented along the length of the CCA and proceeds craniocaudal from the base of the neck; the carotid artery is explored up to the bifurcation, documenting any pathologic wall abnormalities and lumen diameter, in addition to measuring the IMT (see Section 6.4). The carotid bifurcation is systematically examined, followed by the ICA and the ECA. The proximal and distal segments of the ICA are evaluated in detail. Spectral and colour Doppler analyses are performed on both the CCA and the ICA. To ensure accurate velocity measurements, blood flow is sampled from the proximal, middle, and distal segments of the ICA. These measurements should be obtained from a longitudinal plane with a Doppler angle of 60° to the vessel walls. Angle correction is applied within the sample volume to optimise accuracy. A Doppler angle between 30° and 60° is recommended where feasible (see Section 5.2).

Colour Doppler is used to identify areas of interest and to detect the presence of aliasing, which guides the placement of the Doppler sample volume cursor. Spectral Doppler is then recorded, ensuring sampling at the highest velocity and documenting any post-stenotic turbulence that may be present. The examination then proceeds with spectral and colour Doppler analysis of the following vessels: the ECA, the brachiocephalic artery, and the proximal subclavian arteries (right and left). On a deeper, more lateral scanning plane, the vertebral arteries and their respective intertransverse segments are evaluated. This includes documenting spectral and colour Doppler analysis of the artery’s origin, as well as recording proximal (V0 and V1), intermediate (V2), and distal (V3) flow. Particular attention is given to assessing flow direction. The V4 segment can be evaluated via an occipital window using a convex or sectorial transducer. The sample volume should be kept as small as possible, except in cases of suspected occlusion. In the presence of stenosis, sampling is performed proximally, at the site of the minimal residual lumen, and distally to the stenosis. Velocity data, when combined with a correct morphological assessment of stenosis (both in terms of diameter and area), serve to further define the degree of stenosis. Documentation of stenotic pathology should include both morphological and flowmetric data. Morphological data should detail plaque characteristics, while flowmetric data should report the percentage of stenosis or, in the case of vertebral arteries, any flow direction anomalies (e.g., subclavian steal syndrome). For patients with a history of endarterectomy or stenting, the morphological and flow characteristics must be described and adjusted as appropriate. All acquired data must be adequately documented and recorded. The examiner is responsible for reviewing the data to ensure the evaluation is comprehensive and sufficient for generating a final report. Any additional findings related to adjacent structures, such as thyroid nodules or lymph nodes, should also be described and documented.

### 6.4. Carotid Intima-Media Thickness

Carotid IMT serves as a key marker for assessing atherosclerotic risk. During a two-dimensional ultrasound examination, the IMT appears as a distinct double line. This measurement corresponds to the distance between two specific acoustic interfaces: the lumen-intima boundary and the media-adventitia boundary [95,96]. The IMT is considered elevated when it exceeds 0.9 mm or the 75th percentile for a given age, sex, and ethnicity, based on normative tables [95,96].

According to the literature, IMT should be measured in specific groups of individuals [95,96]:

(1) Individuals at intermediate cardiovascular risk, where IMT can be used as a risk modifier [96];
(2) Patients at high cardiovascular risk with complex clinical profiles, including those with familial hypercholesterolemia [97], autoimmune diseases, or those receiving cholesterol-elevating treatments [98];
(3) Individuals with a family history of early-onset cardiovascular disease [96];
(4) Individuals younger than 60 years with severe cardiovascular risk factors [96], including women with at least two documented risk factors [96].

The clinical role of IMT has undergone a significant evolution in recent years. No longer considered a standalone parameter for cardiovascular risk assessment, IMT is now utilised as a diagnostic tool to identify the presence of atherosclerotic plaques. An IMT value greater than 1.5 mm is considered a definitive indicator of these atherosclerotic formations [99]. Although IMT retains its relevance, direct carotid plaque evaluation is the preferred method for overall cardiovascular risk stratification [86]. When a carotid atherosclerotic plaque is detected, the IMT measurement should not be reported unless specifically requested. If a plaque is located in the region typically used for IMT measurement, its dimensions must be included in the plaque assessment [95].

We recommend a standardised IMT measurement protocol similar to the one described in the ELSA-Brasil study [100]. The examination is performed using two-dimensional (B-mode) imaging, with a 7–12 MHz transducer frequency and no magnification. Image quality is optimised by adjusting the gain, setting the focal point at the artery, and selecting a depth of 3.0–4.0 cm. The probe is positioned longitudinally to visualise the distal CCA and the bulb. Images are acquired via anterior, posterior, or sternocleidomastoid muscle windows. The artery must be horizontally aligned within the imaging sector to ensure optimal visualisation of the intima-lumen interface. The IMT measurement is taken on the posterior wall of the CCA, on a straight, plaque-free segment of at least 10 mm, located at least 5–10 mm from the carotid bulb. For accurate measurement, the transducer must be in a lateral position, perpendicular to the ultrasound beam; multiple insonation angles are not recommended.

**Figure 2:**
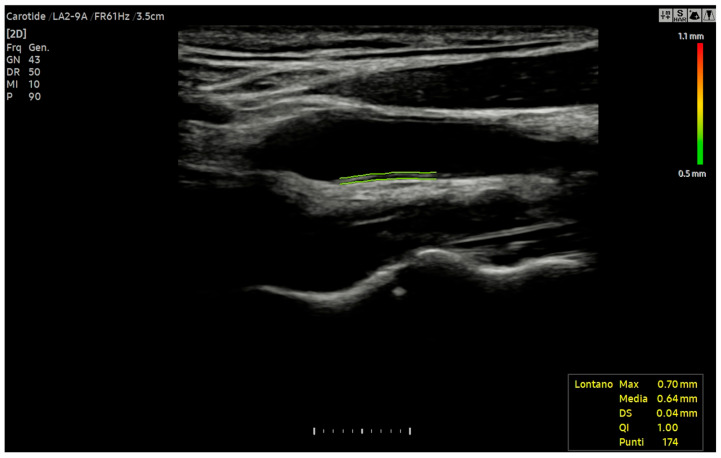
Automatic IMT measurement of far walls of the left common carotid artery using Auto IMT™ software (RS 85, Samsung Medison diagnostic ultrasound system, version 1.05, SAMSUNG MEDISON CO., LTD., 3366, Hanseo-ro, Nam-myeon, Hongcheon-gun, Gangwon-do 25108, Republic of Korea).

To ensure a stable and reproducible value, IMT is measured during the end-diastolic phase, corresponding to the peak of the ECG’s R-wave. This timing is critical as the carotid diameter reaches its minimum in diastole, reducing the influence of physiological variations [101,102]. The adoption of this standardised protocol also facilitates the comparability of results between different studies and centres. For enhanced reliability, it is recommended to obtain multiple measurements (at least three per side) and report the average value. The use of automated or semi-automated software, commonly available on modern ultrasound machines, is the preferred methodology for this procedure [103] (Figure 2).

Manual point-to-point measurement, while less reproducible, is considered an acceptable alternative if the equipment lacks the functionality for automated IMT measurement, provided that technical recommendations are strictly observed [103]. To obtain a representative average IMT value, a minimum of five measurements should be acquired for each side. It is essential to exercise meticulous care in positioning the cursor on the intima-lumen and media-adventitia interfaces to prevent the overestimation of values [103]. Upon data collection, the average values should be compared against established reference values from the normative tables of the ELSA-Brasil [100], CAPS [104], or MESA [105] studies. The selection of the appropriate reference table is contingent upon the patient’s sex, age, and ethnicity. The final report should document the average IMT value for each side in millimetres. Furthermore, the conclusion must indicate whether the value falls above or below the 75th percentile and specify which reference table was utilised, including its bibliographic citation [100,104,105].

### 6.5. Carotid Plaque

#### 6.5.1. Definition

The conventional definition of a carotid plaque is the presence of a localized protrusion of the vessel wall into the lumen measuring ≥1.5 mm, or a focal intimal-medial thickening exceeding 50% of the surrounding area. It is crucial to note, however, that advancements in ultrasound technology now enable the identification of parietal lesions—such as calcifications—that may be smaller than this threshold. Consequently, in addition to the previously described criteria, any focal thickening considered atherosclerotic and extending into the lumen of a carotid artery segment should be classified as a carotid plaque [86].

#### 6.5.2. Methods of Plaque Quantification

The quantification of a carotid plaque should be performed by measuring its maximal thickness or height. To ensure accuracy and prevent potential underestimation or overestimation from off-centre scanning, it is recommended to measure the thickness of the carotid arterial plaque in both the short-axis and long-axis views. The measurement commences by placing the calliper at the adventitial-medial layer (consistent with the manual IMT measurement protocol) and extending it into the centre of the lumen. The highest plaque thickness (or height) obtained from either the short- or long-axis view, along with the specific carotid segment, should be documented in the final report. Additionally, another reported quantification methodology is plaque area, which is calculated by manually tracing the plaque in its most prominent view and largest region. This approach offers a more comprehensive assessment of the atherosclerotic burden, particularly for large, eccentric plaques, and has demonstrated good performance in evaluating atherosclerosis-related outcomes [106]. However, its widespread clinical use is constrained by several limitations, including operator variability, imaging plane dependence, and the presence of calcifications. The carotid plaque score serves as a semi-quantitative ultrasonographic index for assessing the atherosclerotic burden within the carotid arteries. This score is determined by summing the number of carotid segments in which a plaque is detected, from a total of six possible sites: the CCA, the carotid bulb, and the ICA on both the right and left sides. The score ranges from 0 to 6. It is important to note that regardless of the number of individual plaques found within a single segment, that segment contributes only one point to the final score. Although this score has been established as a robust predictor of ischemic stroke and major adverse cardiovascular events [107], particularly for scores exceeding 3, it does not provide critical details regarding plaque size and extension. Consequently, we recommend that this measurement method be reserved for research purposes.

Over the years, 3D-US technology has significantly improved plaque assessment. It allows for a detailed visualisation of plaque geometry and surface, as well as a more precise evaluation of stenosis. While various 3D equipment and techniques are available (see Section 5.6) no single technology is currently recommended. Future research is needed to help standardize 3D-US plaque assessment.

#### 6.5.3. Ultrasound Characteristic of Plaque

Ultrasonographic characteristics of carotid plaque, obtained from a B-mode scan, allow us to estimate its composition. This information is important because plaque composition is linked to its instability and the risk of future vascular events [108]. Both the Gray–Weale classification [109] and the grey-scale median (GSM) [110] are used to evaluate plaque echogenicity, but they are based on fundamentally different principles. The modified Gray–Weale score [111] is a widely used semi-quantitative method that employs a visual classification system for carotid plaques. Based on direct visual inspection by the operator, the score categorizes plaques into five distinct types: (1) uniformly echolucent, (2) predominantly echolucent with less than 50% echogenic components, (3) predominantly echogenic with less than 50% echolucent components, (4) uniformly echogenic, and (5) plaques that are not accessible for evaluation due to extensive calcification. Plaque echogenicity should be standardized using three reference structures:

- Anechoic (no echoes): Use the blood within the vessel.
- Isoechoic (same echoes): Use the sternocleidomastoid muscle.
- Hyperechoic (bright echoes): Use the adjacent transverse apophysis of the cervical vertebrae or the bright far wall media-adventitia interface [112,113].

Plaque echogenicity is linked to the risk of future cerebrovascular events. Plaques of types 1 and 2 are associated with a higher risk [114,115].

A three-year follow-up study on 297 symptomatic patients revealed that plaque echogenicity at baseline is a significant predictor of future cerebrovascular events. Specifically, TIA or strokes occurred in 51% of patients whose plaques were either hypo- or anechoic, in stark contrast to the 4.4% incidence observed in patients with hyperechoic plaques [116].

The GSM value is a measure of a plaque’s overall echogenicity, calculated as the median grey value of all pixels within the plaque image on a B-mode scan. Recent improvements in pixel analysis and colour mapping have increased its accuracy [117,118]. How to evaluate the GSM: (1) Acquire an adequate image of the plaque. (2) Delimit the plaque area using image analysis software. (3) Normalise the grey scale: set the blood in the vessel lumen to “0” (black) and the arterial adventitial wall to “255” (white). (4) The software then analyses the pixels within the plaque to calculate the median grey scale value. A low GSM (dark) indicates a high-lipid or hemorrhagic (unstable) plaque, while a high GSM (bright) suggests a fibrotic or calcified (stable) plaque. A GSM value less than 25 is specifically associated with an increased risk of stroke [119]. According to the GSM method, plaques are categorised into five prevalent types based on their echogenicity.

Type 1 is defined as a uniformly hypo-anechoic plaque with hyperechoic areas occupying less than 15% of the total plaque area.

Type 2 is predominantly hypo-anechoic, with a hyperechoic component ranging from 15% to 50%.

Type 3 is predominantly echogenic, with hyperechoic areas occupying 50% to 85% of the plaque.

Type 4 is a uniformly echogenic plaque where hyperechoic areas exceed 85%.

Type 5 represents a distinct category of plaques that cannot be fully assessed due to an acoustic shadow created by a calcified surface covering more than 15% of the plaque.

The Gray–Weale scale is quicker but more subjective in clinical practice, so the GSM is more dependable for assessing clinical risk and tracking plaque changes.

As previously mentioned, CEUS (see Section 5.5) can assist in identifying high-risk plaque, enabling a more precise assessment.

Ultrasound elastography has shown encouraging results for evaluating carotid plaque vulnerability. This technique, which directly measures tissue stiffness, has proven helpful in differentiating stable carotid plaque from vulnerable ones, confirming its clinical potential to enhance stroke risk stratification. However, further research is needed to validate this method [120,121,122,123,124].

The assessment of carotid plaque surface morphology constitutes a critical parameter, as irregularity or ulceration signifies a substantial vulnerability factor linked to neurological symptoms [108]. Plaque surface morphology is classified into three distinct categories: smooth or regular, irregular, and ulcerated. A “smooth” plaque is defined by a uniform luminal profile, whereas an “irregular” surface is characterised by a variation ranging from 0.3 mm to 0.9 mm [125]. The most widely adopted criteria for defining plaque ulceration in ultrasound are based on three key characteristics: a crater measuring at least 2 mm in both length and depth; a well-demarcated posterior wall on B-mode; and internal flow reversal on colour Doppler [113]. A more recent criterion, however, posits that ulceration can be diagnosed irrespective of size, provided there is evidence of a cavity on the plaque surface with lower echogenicity than the adjacent intimal plaque border on B-mode [126]. 3D Doppler US allows more precise evaluations of the spatial representation of the plaque, particularly its volume, area, and surface (see Section 5.6). 3D Doppler US enables a more precise evaluation of a plaque’s spatial representation, including its volume, area, and surface. Plaque progression along the vessel has been estimated to be 2.4 times faster than wall thickening. Consequently, plaque area and volume, as calculated by 3D US, may serve as superior predictors of future rupture compared to diameter-reducing stenosis [127,128,129,130].

#### 6.5.4. Ultrasound Evaluation of Carotid Stenosis

DUS is a highly accurate method for detecting stenoses greater than 50% and 70% when compared to angiography. This examination has a positive predictive value of over 90% and a specificity of over 85% [131].

**Figure 3:**
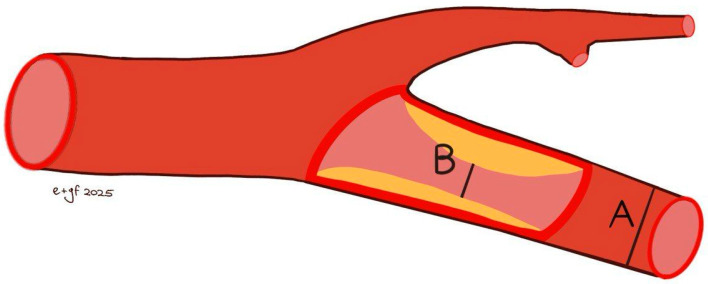
NASCET methods [(B − A)/B × 100% stenosis] for measuring the degree of carotid stenosis. B: residual diameter at the stenosis point; A: distal diameter of the internal carotid.

**Figure 4:**
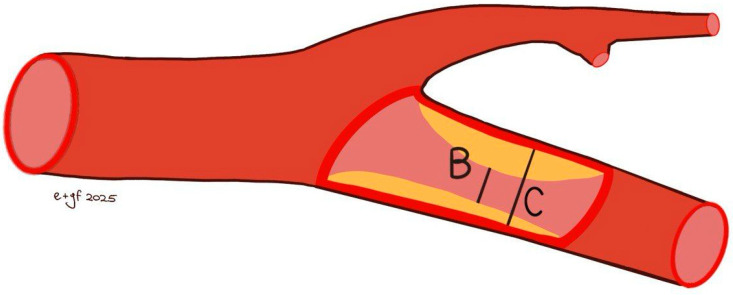
ECST method [(C − B)/C × 100% stenosis] for measuring the degree of carotid stenosis. C: estimated diameter of the interna carotid; B: residual diameter at the stenosis point.

A comprehensive colour Doppler ultrasound examination for carotid stenosis, particularly of the ICA, incorporates all standard ultrasonographic applications. This includes B-mode imaging, colour imaging, and spectral flow analysis with pulsed Doppler, which are all used to obtain multiple observations during the assessment. Each of these modalities provides varying indications based on the degree of stenosis and plaque characteristics. Therefore, a multiparametric evaluation is essential for the most accurate assessment of the actual pathology. B-mode ultrasound is currently the best method for visualising IMT and minor plaques, as it provides images of the vessel wall itself rather than just the blood column. While moderate pathologies are easily visualised on both longitudinal and transverse planes, obtaining clear B-mode cross-sectional images in severe diseases is more challenging due to potential acoustic shadowing and other artefacts. Colour Doppler is useful for the immediate assessment of flow direction and for indicating hemodynamically significant stenosis through aliasing. However, it is not reliable for precise diameter or area measurements because of the variability in parameter control settings and it does not provide accurate estimations of flow velocity. Conversely, Colour Doppler is crucial for indicating the exact positioning of the sample volume needed for accurate flow velocity estimations. Thus, the more severe the stenosis, the more critical the hemodynamic criteria become [132]. At the site of maximum stenosis, an increase in peak systolic velocity (PSV) is detected. A hemodynamic effect is achieved when both pressure and flow volume decrease in the post-stenotic segment. The degree of stenosis is defined as the percentage reduction in the vessel lumen at the stenosis site relative to the vessel’s original diameter. This definition, however, is not universally comprehensive, as validation studies of carotid stenosis criteria have focused almost exclusively on the bifurcation and extracranial ICA, typically in cases of atheromatosis. Consequently, there are no standardized criteria for detecting stenosis in other cerebrovascular segments. The two primary criteria used to assess the degree of stenosis are: (1) the North American Symptomatic Carotid Endarterectomy Trial (NASCET) criterion, which compares the residual diameter at the stenosis site to the distal diameter of the ICA, and (2) the European Carotid Surgery Trial (ECST) criterion, which compares the residual diameter at the stenosis point to the estimated original diameter of the ICA [133,134,135] (Figure 3 and Figure 4).

Both NASCET and ECST measurement methods are feasible with B-mode imaging, and the specific method used should always be documented. These methods are susceptible to errors, including vessel malalignment, operator variability, and the presence of severe calcifications. Furthermore, the adoption of the NASCET method is limited in cases of high bifurcation, severe tortuosity, and extremely long plaques. The ECST method typically results in higher degrees of stenosis. The conversion between NASCET and ECST percentages has been well-established through the following formulas: NASCET % = (ECST-40) %/0.6 and ECST % = 40 + (0.6 × NASCET %) [136]. The relationship between ECST and NASCET percentage of stenosis is illustrated in Table 1.

For extreme degrees of stenosis, the following terms can be used:

- Sub-occlusion (90–95% stenosis): The stenosis is segmental and tight at the origin of the ICA from the carotid bifurcation, but the post-stenotic lumen resumes, albeit reduced.
- “Near-occlusion”: Partial or subtotal collapse caused by inadequate filling of the lumen in the ICA over a long segment from its origin to the carotid bifurcation towards the cranial base; also known as “string-sign” on instrumental evaluations. Notably, CTA is especially useful in distinguishing near occlusion from sub-occlusion.
- Several CTA parameters have been established: no distal vessel collapse, including (1) residual lumen of 1.3 mm; (2) ipsilateral distal ICA diameter of 3.5 mm; (3) ratio of ipsilateral distal ICA diameter to contralateral ICA of 0.87; and (4) ratio of ipsilateral distal ICA diameter to ipsilateral ECA diameter of 1.27 [137]. More recently, improved prognostic discrimination has been demonstrated with the combination of a distal ICA diameter of 2 mm and an ICA diameter ratio of 0.42 [138].
- Occlusion: It can be segmental at the origin of the ICA or entire throughout the extracranial ICA. In such cases, aside from the absence of colour Doppler, the PW Doppler analysis at the CCA may show a significant reduction in EDV compared to the contralateral CCA [139] or a “knocking” waveform pattern (low PSV, decreased, absent, or reversed diastolic flow, high resistance waveform pattern).

In addition to morphological data, velocimetric data are crucial for the quantitative assessment of stenosis. Numerous studies demonstrate the correlation between velocity and the degree of stenosis, examining the accuracy and reliability of this classification criterion, which has consequently become a globally accepted standard [86]. The most important parameter is the PSV within the stenosis, representing the highest blood flow velocity during systole, measured by placing the Doppler sample volume at the site of maximum stenosis, where aliasing is detectable in colour mode. According to the most widely used criteria, if the PSV exceeds 125 cm/s, a stenosis greater than 50% is diagnosed in the presence of a sonographically visible plaque in the ICA. PSV exceeding 230 cm/s indicates stenosis of ≥70% [109]. However, due to the lack of a universally accepted and validated consensus, different velocity values may be used. For example, the value reported by the Deutsche Gesellschaft fur Ultraschall in der Medizin (DEGUM), which proposed 200 cm/s for 50% ICA stenosis according to the NASCET method [140]. It is important to note that PSV measurement has several limitations, such as Doppler angle, stenosis morphology, collaterals, nearly occluded arteries, and atrial fibrillation. Additionally, PSV cannot distinguish between severe (70%) and very severe stenosis (between 70 and 80–90%). In such cases, the systolic peak downstream may be lowered and rounded with poor systolic-diastolic differentiation, known as “pulsus tardus et parvus”. Moreover, assessing the extent of reduction in post-stenotic flow aids in distinguishing between different stenosis grades; some studies indicate that a post-stenotic PSV < 50 cm/s and <30 cm/s characterise 80% and 90% stenosis, respectively [132]. In cases involving plaque with an acoustic shadow (i.e., circumferential calcified plaque), the PSV cannot be measured at the site of the most severe stenosis. Some authors suggest evaluating the acceleration time (ACT) ratio, calculated as the ratio between ACT at the distal ICA and ACT at the ipsilateral CCA [139]. A cutoff value of 1.5 determines a NASCET stenosis rate of >65% with a sensitivity of 90.0% and a specificity of 93.5% [141]; another study found that an ACT ratio cutoff of 1.31 had 94.5% sensitivity and 91.0% specificity in diagnosing stenosis of ≥50% [142].

To obtain a more precise classification of stenosis, in addition to PSV sampling, other parameters should be evaluated, such as the end-diastolic velocity (EDV), the ratio between PSV at ICA and the CCA, and the St Mary’s ratio (PSV ICA to EDV CCA ratio [143]. In particular, if the stenosis is below 50%, the ICA/CCA PSV ratio will be less than 2.0 (even in case of a slight increase in PSV at the stenosis level); for stenosis values between 50 and 69%, the ICA/CCA PSV ratio will be between 2.0 and 4.0; and finally, for stenosis values equal to or greater than 70%, it will be greater than 4.0 according to NASCET criteria [144]. Recently, a new parameter, the PSV ICA/ICA ratio (PSV sampled at ICA stenosis and distal to stenosis), has shown comparable correlations with CTA in evaluating carotid artery stenosis [145]. Table 2 shows the multiparametric evaluation we recommend for carotid stenosis assessment.

#### 6.5.5. Post-Operative Checks

Approximately 5–10% of patients may develop restenosis within 5 years after Carotid Endarterectomy (CEA). This restenosis is often caused by excessive scarring at the suture site, in addition to the progression of atherosclerotic disease. Restenosis following Carotid Artery Stenting (CAS) can range from 10% to 20% or more, depending on patient characteristics and the specific technique employed. It is primarily due to neo-intimal proliferation within the stent and can be influenced by factors such as the type of stent and placement techniques [147]. Distinguishing between intimal hyperplasia (more frequently observed within the first 6–24 months) and atherosclerotic restenosis (typically occurring after 2–3 years) is crucial for guiding follow-up strategies and therapeutic decisions [148,149]. From a sonographic perspective, intimal hyperplasia is characterized by a regular, typically concentric, and hypoechoic thickening of the vascular wall, associated with early myointimal proliferation. Conversely, atherosclerotic restenosis often presents as an eccentric lesion that may contain mixed components (fibrous, lipid-rich, or calcified). It may also be associated with intraplaque neovascularization, which can be detected using advanced imaging techniques like CEUS [150,151,152]. While a progressive increase in PSV over time may suggest the development of significant restenosis, regardless of its underlying etiology, Doppler velocity measurements alone are insufficient for definitive differentiation. A comprehensive interpretation, considering both the clinical and morphological context, is essential. This includes applying the principle of continuity by comparing in-stent PSV with velocities measured in adjacent, unstented segments to minimize the risk of overestimation from Doppler artifacts [151].

##### Carotid Endoarterectomy

A review of current literature indicates that there are no statistically significant differences in early restenosis rates among the various surgical techniques used for CEA, including patch angioplasty (utilizing materials such as polytetrafluoroethylene or bovine pericardium) and eversion endarterectomy [137,153]. Regardless of the specific technique used for open surgical interventions, intraoperative morphological control at the conclusion of an endarterectomy is crucial for preventing or reducing postoperative complications related to surgical technical errors. After a few years, restenosis may occur due to the recurrence of atherosclerotic disease, particularly in patients who have not corrected their risk factors. The incidence of restenosis within 1 to 2 years varies from 9% to 33% [154]. Therefore, it is advisable to perform regular instrumental follow-up to monitor for any pathological progression or the development of contralateral disease [155].

Prospective studies have shown that ultrasound is reliable compared to angiography, enabling the detection of technical defects that require immediate reoperation and establishing a baseline for follow-up monitoring [156]. As distinct from stenting, the absence of an intraluminal prosthesis after CEA generally results in fewer Doppler artifacts, which makes ultrasound interpretation more reliable [157,158]. However, the presence of prosthetic patches, irregular suture lines, or post-surgical angulation can still induce localized hemodynamic disturbances, necessitating careful Doppler waveform analysis [153]. The Doppler ultrasound parameters evaluated during the follow-up of a CEA are the same as those assessed in native carotid stenosis, including PSV in the ICA and the PSV ICA/CCA ratio. However, the cut-offs vary due to the structural changes in the vessel caused by the intervention; indeed, the ultrasound criteria for diagnosing re-stenosis after CEA are: ICA re-stenosis > 50% → PSV 213 cm/s; PSV ICA/CCA ratio 2.25. ICA re-stenosis > 70% → PSV 274 cm/s; PSV ICA/CCA ratio 3.35 [153].

The Society for Vascular Surgery (SVS) guidelines recommend a structured duplex ultrasound surveillance protocol: a baseline examination within the first month, followed by biannual follow-up for two years, and then annual evaluations if no abnormalities are detected [159]. In contrast, the 2023 ESVS guidelines do not define specific surveillance intervals based on surgical technique. Instead, they emphasize the importance of individualized follow-up that considers the patient’s overall risk profile, contralateral disease, and disease progression [137].

##### Carotid Artery Stenting

Duplex ultrasound surveillance after CAS aims to assess the technical success of the procedure, detect recoil phenomena or stent underexpansion, and identify residual stenosis or postprocedural complications [160].

The recommended follow-up schedule for patients undergoing CAS is generally similar to that applied after CEA. However, one key consideration is the importance of performing an early baseline duplex ultrasound examination, due to the high hemodynamic variability often observed in stented carotid segments. Acquiring these initial Doppler measurements provides a valuable reference point for future comparative assessments during longitudinal surveillance [161].

This close US surveillance allows for early detection of severe restenosis, enabling timely reintervention and the prevention of cerebral ischemia events. Diagnosing restenosis after CAS with ultrasound is challenging, as the stent itself increases blood flow velocities [162]. The main types of carotid stents currently used include open-cell stents, closed-cell stents, and more recently, dual-layer or micromesh stents (also referred to as low-profile micromesh) with a two-layer design.

It is important to recognize that stent architecture can influence not only flexibility and vessel conformability, especially in tortuous anatomies, but also flow dynamics and Doppler ultrasound interpretation following the procedure.

Several studies have shown that after CAS, Doppler velocities, particularly PSV and the ICA/CCA ratio, are typically higher than expected when compared to non-stented vessels, even in the absence of significant anatomical stenosis. This phenomenon is primarily attributed to the mechanical rigidity introduced by the stent, which alters the normal flow profile.

In a cohort of 141 CAS patients with well-patent angiographic results, Pierce et al. demonstrated that average PSV values were significantly higher in closed-cell stents (mean 122 cm/s) than in open-cell stents (mean 95.9 cm/s), with an odds ratio of 2.2 for presenting with abnormally high velocities [163]. These findings suggest that Doppler criteria derived from native carotid arteries may require adjustment based on stent design.

Similarly, Schäberle [151] reported that closed-cell stents tend to produce higher PSV values than open-cell models (115 vs. 93 cm/s). The author emphasized that many studies fail to account for stent design as a variable, despite evidence that design-induced rigidity can significantly influence post-procedural velocities. Schäberle also highlighted the value of obtaining an early post-implantation Doppler baseline to facilitate future comparisons.

Regarding dual-layer or micromesh stents, Sýkora et al. [164] observed in a median 24-month follow-up that the incidence of severe in-stent restenosis (≥70%) was significantly higher in patients with dual-layer stents (13.3%) compared to those with single-layer stents (3.4%, *p* = 0.01). The dual-layer group also had a higher rate of reintervention, suggesting that micromesh stents may be more prone to in-stent restenosis, at least in observational clinical settings.

From a sonographic perspective, the use of complex stent structures (e.g., tightly meshed or dual-layer designs) can introduce artifacts that complicate Doppler interpretation, such as ultrasound wave reflection, scattering, flow profile distortion, local turbulence, and overshoot or aliasing phenomena.

These distortions may lead to overestimation of stenosis severity if standard Doppler thresholds derived from native arteries are applied without accounting for the hemodynamic effects of the stent.

The main parameters to monitor with US are

- Presence and severity of stenosis at the treatment site (in-stent).
- Progression of untreated contralateral stenosis [160,165].

To improve diagnostic accuracy and reduce the risk of misinterpretation, we recommended recording baseline Doppler velocities immediately after stent implantation. Most studies use the following PSV thresholds:

- Restenosis >50%: PSV > 220 cm/s; PSV ICA/CCA ratio > 2.5.
- Restenosis >70%: PSV > 300 cm/s; EDV > 90 cm/s; PSV ICA/CCA ratio > 3.8 [158,166].

However, a sub-analysis of the international carotid stenting study, which compared DUS-derived PSV with CTA in patients with restenosis after CAS, found lower cut-off values: restenosis > 50% → PSV > 125 cm/s and PSV ICA/CCA ratio 1.5 [167].

We recommend evaluating the following US parameters to characterise restenosis after CAS:

- Patency of the ICA.
- Patency of the ECA.
- Presence of stenosis in the stented segment.
- Presence of stenosis (new atheroma, hyperplasia, thrombus) inside the stent (in-stent restenosis).
- Presence of stenosis upstream or downstream of the stent.
- Presence of parietal thrombus.
- Adhesion of the stent to the vessel wall.
- Presence of kinking of the internal carotid at the end of the stent (caused by different compliance between the stent and the carotid).
- Migration of the stent.
- Integrity or breakage of the stent.
- Any complications related to a previous endarterectomy (patch detachment, etc.).

Additionally, in patients with complex, long, or high-density metallic stents, rigid application of standardized Doppler velocity thresholds should be avoided. Instead, an individualised approach is advised, based on clinical progression and serial duplex ultrasound findings [151].

The surveillance timing with ultrasound following CEA and CAS are summarised in Table 3 while the main duplex ultrasound velocity criteria for carotid restenosis after CAS and CEA are summarised in Table 4. 

### 6.6. Carotid Dissection

Arterial dissection is a pathological process in which the intima, the innermost layer of an artery, becomes separated from the media because of an intimal tear. The detached intimal flap typically exhibits a characteristic fluttering or mobile motion within the true arterial lumen. The presence of this intimal tear facilitates the formation of a false lumen into which blood can flow and/or a thrombus may develop. Hemodynamics within the false lumen can be described as a ‘cul-de-sac’ when blood enters and exits through the same tear, or as a ‘double-barrel’ flow when blood exits via a second tear, which may be situated either distally or proximally to the initial entry point [168]. Clinical presentation varies from incidental findings in asymptomatic patients to headache or neck pain, Horner’s syndrome, tinnitus, and cranial nerve paralysis [169].

In non-traumatic cases, hypertension is the most common predisposing factor for carotid artery dissection. However, it should be remembered that some conditions such as Ehlers-Danlos syndrome and Marfan syndrome may increase vessel wall fragility [169].

Carotid ultrasound is the initial imaging step to detect carotid artery dissection. B-mode imaging may reveal a tapered lumen with a characteristic string sign and a floating intimal flap.

Colour Doppler analysis may reveal bidirectional low-amplitude flow. Due to the false lumen’s mass effect, Doppler spectra might display a high-resistance pattern with delayed systolic acceleration, reduced diastolic flow, and eventually bidirectional systolic flow with minimal diastolic flow. Flow direction within the false lumen can be antegrade, retrograde, or bidirectional when the sample volume is placed inside it. Dissection extending to the CCA generally indicates an aortic dissection. Angiography, the gold standard for confirming a diagnosis after ultrasound, is an invasive procedure. CT and MRI can provide reliable diagnoses [170,171].

Blood flow, low bidirectional flow, or low post-stenotic velocities may be observed at the atlas loop, which is a common site of dissection. Direct ultrasound visualisation of intramural haematoma is rare; further imaging, such as MRI with fat-saturation sequences or cerebral arteriography, should be carried out when vertebral artery dissection is clinically suspected, even if ultrasound results are conclusive [171].

### 6.7. Carotid Morphological Anomalies

**Figure 5:**
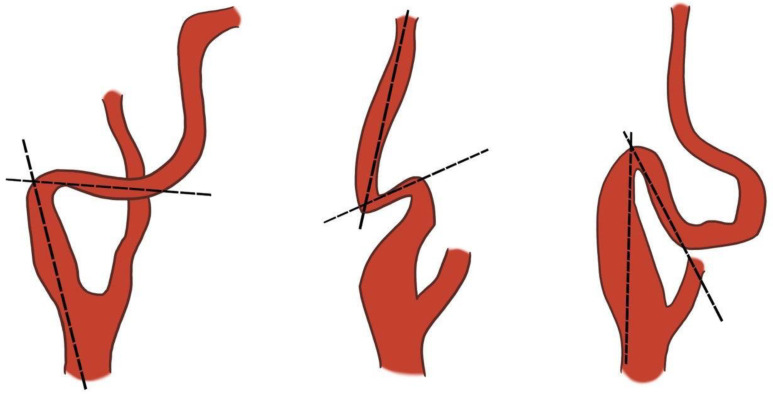
Metz’s classification of carotid kinking. Dotted lines drawn along the axis of the internal carotid artery show the curvature of the vessel less than 90° (**left**) or 60° (**centre**) or 30° (**right**). Adapted and modified from Metz H et al. [175].

Morphological anomalies of the carotid artery are common in the general population, occurring in approximately 10% to 45% [172]. About 75% are located 2–4 cm proximal to the carotid bifurcation but can also be observed more distally. These anomalies are classified into three types: tortuosity, coiling, and kinking [173]. Tortuous carotid arteries are typically asymptomatic, whereas coiling and kinking may be associated with more haemodynamic abnormalities and flow impairment. Coiling involves a complete 360° rotation of the arterial tract, while kinking is defined as a morphological anomaly characterised by an acute angulation (not exceeding 90°) [174]. According to Metz, the kinking curves are classified based on the angle:

According to Metz, the kinking curves are classified based on the angle:

Metz 1 or mild kinking (<90°), Metz 2 or moderate kinking (<60°), and Metz 3 or severe kinking (<30°), Figure 5 [175].

Morphological anomalies are more common in the ICA, and it can be challenging to follow the vessel as it changes planes and curves. Colour mode is essential for tracking tortuous arteries. Notably, the presence of turbulent flow and high velocity on Doppler does not strictly indicate the degree of stenosis, as Doppler velocity measurement depends on the examination angle. Therefore, there is no specific velocity threshold for tortuous vessels due to the variety of angulations that may occur. Di Pino et al. assessed the ratio between the maximum velocity recorded at the kink or coiling level and two centimetres proximal to the carotid bulb. The authors considered a ratio < 1.5 as indicative of <50% stenosis; a ratio > 3.2 predicts > 60% stenosis, while a ratio > 3.3 predicts > 70% stenosis [174]. In cases of significant stenosis, post-stenotic turbulence may be observed with a lowered and rounded systolic peak downstream.

We recommend recording Doppler waveforms at the point of the tightest curve after aligning the angle cursor parallel to the artery walls (30–60°). Additionally, in cases involving coils, which can be in different planes, imaging should be performed from various axes, planes, and neck angles. Using a convex probe with frequencies between 2 and 5 MHz may assist in capturing a complete image of the carotid alteration and achieving greater depth.

### 6.8. Miscellaneous

#### 6.8.1. Carotid Fibromuscular Dysplasia

Fibromuscular dysplasia (FMD) is a rare, idiopathic condition affecting medium- and small-sized arteries, characterised by architectural anomalies and non-inflammatory cellular proliferation. It most often involves the extracranial segments of the carotid and vertebral arteries, referred to as cerebrovascular FMD (C-FMD). Although FMD can present at any age, it mainly occurs between 43 and 53 years, with a marked female predominance (82–95%). Men tend to present with more severe forms of the disease. The renal arteries are the most affected, followed by the cerebrovascular circulation. Multifocal involvement is observed in 71.9–76% of cases, with the prevalence of a multivessel subtype ranging between 31.2% and 55.1% [176].

FMD is characterised by a concentric or tubular narrowing that can occur in any segment of a vessel. The concentric type is typically less than 1 cm in length, while the tubular type is 1 cm or more [177]. In contrast, the multifocal subtype presents with alternating areas of stenosis and dilation, creating the characteristic ‘string-of-beads’ appearance, which typically affects the mid and distal portions of the involved vessels. Multifocal FMD is the most common type, accounting for 90% of cases, followed by the focal variant, which represents the remaining 10%. Although FMD is primarily a stenotic disease, its imaging spectrum also includes aneurysms, tortuosity, and dissection [178].

C-FMD often remains clinically asymptomatic and is typically diagnosed incidentally during imaging studies performed for other indications. The most common symptom, reported in up to 70% of patients, is a chronic headache, which may be related to variations in cerebral blood flow or structural dysfunctions. Another notable symptom is pulsatile tinnitus, a sound synchronised with the heartbeat, which occurs in a variable percentage of patients. The broader spectrum of C-FMD manifestations includes more severe cerebrovascular events, such as TIAs and ischemic strokes [179].

The primary imaging modalities for cerebrovascular C-FMD include

- Angiography: While considered the gold standard, angiography is typically reserved for patients with severe vascular findings that require endovascular intervention. This is because of the associated risk of iatrogenic dissection [176].
- CTA and MRA: These are the most common modalities for the initial diagnosis of C-FMD. They provide detailed images of the cerebral arteries and are effective at highlighting abnormalities like stenosis and aneurysms, often revealing the characteristic ‘string-of-beads’ sign [176,180].
- Carotid DUS: This technique is primarily used in highly specialised centres. It can detect subclinical vascular abnormalities such as tortuosity or altered blood flow in the carotid arteries. Although standardised diagnostic criteria for DUS are currently lacking, abnormal findings may suggest the presence of FMD [176].

Although less sensitive than other imaging techniques, carotid ultrasound can provide valuable diagnostic clues for C-FMD. Key findings include

- Increased Doppler Velocity: Elevated velocity in the distal carotid arteries may indicate stenosis.
- Flow Turbulence: Colour Doppler ultrasound can demonstrate turbulent blood flow, characterised by aliasing, particularly in the distal segment of the ICA.
- “String-of-beads” Sign: Although this sign, which represents alternating arterial lumen narrowing and dilation, is rarely visualised with ultrasound, it is diagnostically significant when seen.

A crucial diagnostic consideration is that FMD primarily affects the mid-distal ICA, whereas atherosclerotic disease predominantly involves the proximal ICA at the bifurcation. Recognising this key difference is essential for accurate diagnosis and image interpretation [180].

##### Carotid Web

Carotid Web (CaW), also known as a carotid diaphragm, is an unusual variant of FMD characterised by a thin, fibrous membrane-like structure. This structure originates from the posterior wall of the internal carotid artery bulb, projecting into the arterial lumen. Although a rare clinical entity, numerous studies have emphasised a strong link between CaW and stroke, particularly in younger patients without other vascular comorbidities [181]. According to research by Kim SJ et al., the prevalence CaW is less than 1% in patients without ischemic stroke [182]. The overall population incidence of CaW remains largely unknown; however, affected individuals are most often young (under 60 years old), female, and of African American descent. Among younger patients with cryptogenic stroke, the incidence of CaW is estimated to be approximately 13% [181,183].

The pathogenesis of CaW remains a subject of ongoing debate. Histologically, CaW is characterised by focal intimal hyperplasia [184], setting it apart from classical FMD (which involves hyperplasia or fibroplasia of the tunica media) and atherosclerotic lesions. Early studies suggested that CaW might be a congenital defect resulting from abnormalities in the development of the brachiocephalic system [185]. This hypothesis is supported by its frequent occurrence in young patients without cardiovascular risk factors. However, a study of 47 paediatric stroke patients found no cases of CaW, suggesting that it may instead be an acquired lesion [186]. Given the observed association between CaW, African American ethnicity, and female sex, it is also plausible that genetic predispositions or hormonal factors contribute to the development of Caw. Further research is needed to clarify the precise pathogenesis of CaW.

CaW can disrupt blood flow and cause stasis between the intima and the carotid wall, promoting thrombogenesis and embolic risk. The presence of a CaW alters blood flow, generating vortices that indicate turbulent flow, thus increasing the risk of thrombosis. Studies using V-flow methods and vectorial analysis have revealed specific hemodynamic patterns in CaW, highlighting parameters such as mean shear stress and oscillatory shear index. Recirculation zones are significantly larger in patients with CaW, and elevated shear stress values suggest an environment conducive to thrombosis. Specifically, high mean shear stress at points of minimal lumen diameter is associated with an increased risk of thrombus formation. A retrospective analysis of 41 patients demonstrated that CaW is associated with greater hemodynamic disturbances than atherosclerotic lesions [187]. Stroke risk is also correlated with CaW size and morphology. Tabibian et al. identified that a length ≥3 mm, an acute angle, and an occupation of >50% of the carotid bulb are associated with increased stroke risk [188].

CaW can be identified using various diagnostic methods, with CTA being the most widely employed technique. This modality provides excellent resolution for visualising CaW, which appear as thin, shelf-like projections from the posterior wall of the internal carotid bulb [189]. Carotid Doppler ultrasound serves as a useful first-line diagnostic tool for assessing CaWs; however, its resolution is inferior to other modalities, such as CTA or catheter angiography, and its reliability in distinguishing CaW from atherosclerotic disease is limited [190].

Distinctive ultrasound features of a CaW include

- Location: CaW is often situated at the proximal ICA and the carotid bifurcation.
- Appearance: CaW appears as a membranous structure protruding into the arterial lumen, without significant fluctuations with blood flow, unlike free-floating thrombi.
- Size: In some patients, the CaW extends over half the artery’s diameter, while in others, it is comparatively short.
- Longitudinal versus transverse views: CaW is more easily visualised in longitudinal views and appears clearer than in transverse sections.
- Cliff-like stenosis: In certain cases, CaW causes an abrupt, cliff-like narrowing of the artery.
- Presence of plaques and thrombi: Plaques are often associated with CaW, and in some cases, thrombi can be observed forming at the acute angle between the CaW and the arterial wall. These features help clinicians improve the diagnostic accuracy of CaW during ultrasound assessments [191].

The extra use of MVFI ultrasound can detect slow blood flow in about 82% of CaW cases, thereby enhancing ultrasound sensitivity. During MVFI ultrasound, CaW appears as a triangular endoluminal defect with a reduced-flow area (nidus) beneath it during systole. This enables precise delineation of the CaW, even when it is not visible using B-mode ultrasound [192].

##### Thin Fluttering Band

Another membrane-like structure that may be casually encountered during carotid US scans is the thin Fluttering Band (TFB). Such a thin structure, quite different from the CaW, is defined as the presence of a hyperechoic fluttering band within the carotid lumen, in the absence of any echographic sign of dissection [193]. According to the literature, TFBs are mostly single (91.5%), with a small percentage being multiple (8.5%). The most common location is the carotid bulb, followed by the ICA and CCA [193]. Several hypotheses have been proposed regarding the nature of TFBs: such structures may represent (1) a vascular remodelling phenomenon in the early stages of atherosclerosis; (2) a sign of arterial intima turnover, reflecting a dynamic change in the vascular wall in response to the mechanical forces of blood flow; or (3) the result of a bacterial biofilm adhering to the vascular wall and contributing to the atherosclerotic process.

The US is the most suitable method for characterising their nature, as other radiographic imaging techniques, such as CTA or MRI, failed to visualise TFBs [193]. On B-mode scan, TFB appears as a thin hyperechoic floating structure within the carotid lumen, visible in both longitudinal and transverse views. M-mode imaging is useful, emphasising the non-congruent movement of TFB relative to the vessel wall, thereby distinguishing them from reverberation artefacts or venous valves (which are thicker than TFB and move in opposite directions [194]. Colour Doppler reveals flow alterations associated with TFB, particularly in the carotid bulb, where the flow appears more turbulent.

#### 6.8.2. Aneurysm and Pseudoaneurysm

Extracranial true carotid aneurysms are rare. The aneurysm is a localised dilatation of the artery involving all three layers of the arterial wall, exceeding 50% of its expected luminal diameter compared to the diameter of the contralateral, unaffected carotid artery, the ipsilateral arterial segment proximal or distal to the aneurysm, and free from aneurysmal changes [195,196]. Some authors define bulb carotid aneurysm as a dilation greater than 200% of the diameter of the ICA or greater than 150% of the diameter of the CCA [197].

The most common location for carotid artery aneurysms is the proximal ICA, followed by the carotid bifurcation [198,199]. The main cause of carotid aneurysm is atherosclerosis, while in some cases they result from infection (mycotic aneurysms), trauma, FMD, and connective tissue disorders [198,199,200].

Carotid aneurysm may be asymptomatic or present with a pulsatile mass in the neck. US is typically the first-line imaging modality, although there is a lack of uniform consensus in the literature regarding US parameters [201]. We recommend that the examination include multiple B-mode images in both longitudinal and transverse planes, measuring anteroposterior and mediolateral diameters, and reporting the methodology used to determine the maximum diameter of the enlarged vessel (inner-outer, outer-outer). The morphology of the aneurysm, whether saccular or fusiform, must be documented [201]. The presence of mural thrombus and echogenicity characteristics should be evaluated [202]. It is also crucial to differentiate between a true aneurysm and a pseudoaneurysm. A true aneurysm is characterised by a localized dilatation of the vessel involving all three vascular layers, which remain intact. In contrast, pseudoaneurysms involve a disruption of the continuity of all three layers of the arterial wall. Pseudoaneurysms often exhibit a different appearance due to their nature, arising from a rupture of the vessel wall and the formation of a sac containing blood outside the vessel. The presence of calcifications within the aneurysm wall suggests that the dilatation is a true aneurysm [201]. In pseudoaneurysm, colour Doppler in transverse scan typically demonstrates a “yin-yang” appearance, with red on one half of the mass and blue on the other, indicating flow into and out of the mass. On PW Doppler, the key feature is a to-and-fro flow pattern, usually with high velocities.

#### 6.8.3. Radiation Effects

Radiation-induced arterial injury results from therapeutic irradiation during the treatment of various tumours, such as those in the head, neck, or upper thoracic region [203]. Vascular damage occurs due to radiation effects on endothelial cells, which are highly sensitive to radiation. Injury to the vasa vasorum in the medial layer of the artery leads to fibrosis, causing a narrowing of the lumen. Patients frequently have a history of radiation exposure many years prior to examination. Typically, these patients lack common cardiovascular risk factors, and the atherosclerotic lesion may be longer and located at atypical sites [204]. Therefore, ultrasound examination should include thorough B-mode imaging of the common carotid arteries, given the high incidence of radiation-induced stenosis. Additional parameters to assess include the echogenicity of the lesion and PW Doppler analysis at the site of stenosis [205].

#### 6.8.4. Carotid Body Tumor

Carotid body tumour, also known as carotid paraganglioma or chemodectoma, is a rare hypervascular tumour located at the adventitia of the common carotid artery bifurcation [206]. Chemodectoma contains many micro-arteriovenous fistulas with rapid intratumour blood circulation. Most carotid paragangliomas originate from parasympathetic tissue, are usually unilateral, asymptomatic, and inactive, with a slightly higher prevalence in women [207]. Additionally, they grow slowly and have a low metastasis rate [208]. Therefore, complete surgical resection is often curative and offers a favourable prognosis when diagnosed early. The most common presentation of carotid paraganglioma is a rubbery, non-tender mass in the lateral neck, below the angle of the jaw. The mass is more mobile in the horizontal plane (Fontain’s sign), may be pulsatile, and a carotid bruit can be auscultated. US can be utilised as an initial screening test. Key findings of carotid tumour on US are

- On B-mode scan, the mass is well-defined, predominantly hypoechoic, and located at the bifurcation of the CCA. Typically, the mass separates the ICA and the ECA, also known as the “lyre sign” [209].
- Colour Doppler reveals high vascularity with multiple flow signals and low-resistance arterial waveforms on PW Doppler. Additionally, elastography may provide further insights into the tumour’s extracellular matrix and stromal component, displaying a soft to mildly stiff profile [210]. CTA and magnetic resonance imaging offer enhanced characterisation of the tumour. For pre-operative planning, axial MRI facilitates assessment of the circumferential contact degree between the carotid body tumour and ICA [211].

### 6.9. Temporal Artery Disease

US is the primary imaging method for diagnosing GCA because it offers high resolution, quick results, low cost, and no ionizing radiation. The most characteristic sonographic feature is the so-called “halo sign”, a hypoechoic, homogeneous, and concentric thickening of the arterial wall that stays non-compressible when pressure is applied with the probe (positive compression sign) [212,213]. This finding indicates inflammatory oedema of the vessel wall, and when seen bilaterally in the superficial temporal arteries (STA) and their branches, it greatly enhances diagnostic specificity, reaching nearly 100% when bilateral [214]. The IMT of the temporal arteries in GCA is usually over 0.4 mm, often between 0.5 and 0.8 mm [212,215]. However, the sensitivity of this sign varies: about 75% in biopsy-confirmed cases but can fall to 20% if inflammation is limited to the perivascular or adventitial layers [216,217]. The average specificity is approximately 83%, increasing to nearly 100% bilaterally [214]. From a technical standpoint, employing high-frequency linear probes (≥15 MHz, ideally 18–20 MHz) is recommended, with optimised settings: Doppler frequency between 7–12 MHz, PRF 2.5–3 kHz, and focal depth around 4 mm [218,219]. The examination should be performed with minimal probe pressure to prevent disappearance of the halo sign and artefact-related changes. It is advisable to examine bilaterally the STAs (common, frontal, and parietal branches) and, in cases of suspected extracranial involvement, also the axillary, occipital, facial, femoral, and popliteal arteries [219,220,221,222,223,224,225]. Notably, the axillary artery typically has an IMT of about 0.6 mm, while thickening of ≥1 mm (especially ≥1.5 mm) strongly suggests vasculitis [215,226].

We recommend assessing the STAs as follows: place the patient in a supine position with the head rotated contralateral to the artery being examined, potentially supported by a pillow. Identify the common branch of the STA in a transverse scan in front of the ear. Then, orient the wave longitudinally, and evaluate the vessel in B-mode and flow-analysis modes (e.g., CDI, Power Doppler, MVFI) up to the bifurcation. Continue the scan along the parietal and frontal branches using colour mode. Confirm vessel thickening by gently compressing with the probe: diseased vessels have walls that are circumferentially thickened and do not compress (Halo sign, see below). Ultrasound also facilitates the detection of stenoses (aliasing and systolic velocity increase ≥2 times compared to adjacent segments) and occlusions (absence of Doppler signal with intraluminal hypoechoic material), as well as the assessment of the main pathological patterns described [227,228]. The compression sign, which reveals the artery’s failure to collapse under pressure, has high interobserver reproducibility and is considered highly specific for GCA [229]. The halo sign generally resolves within 2–3 weeks of corticosteroid therapy but can disappear as early as 2 days or last up to 6 months in some cases [230,231,232]. Consequently, ultrasound should ideally be performed before or immediately after starting treatment. In terms of accuracy, ultrasound has demonstrated comparable sensitivity (63%) to temporal artery biopsy (69%), though with slightly lower specificity (79% vs. 100%) [233]. Due to its ability to evaluate long vascular segments and detect extracranial cases (which may account for up to 40%), it is often more useful in the initial clinical assessment [220]. Therefore, ultrasound remains a valid alternative to temporal artery biopsy [234]. In large vessel vasculitis (LVV), such as extracranial GCA and TAK, ultrasound is becoming increasingly important. In TAK, involvement is usually asymmetric, affecting the subclavian and common carotid arteries without involving the STA [212,218,235].

The typical ultrasonographic sign of TAK is the “macaroni sign”, characterised by diffuse, homogeneous, concentric mid-echoic thickening primarily of the CCA [216]. The normal IMT of the subclavian artery is approximately 0.6 mm; values exceeding 1 mm suggest vasculitis [215,224]. During the active phase, the wall appears hypoechoic; in the chronic phase, it tends to become hyperechoic [224,225]. Ultrasound parameters of GCA-TAK are summarised in Table 5. The use of CEUS enables the assessment of mural neovascularisation as an indicator of inflammatory activity [236,237,238]. However, despite its potential, standardisation is still required, and it does not replace comprehensive imaging modalities (CTA, MRA) for determining the extent of disease [239]. Ultrasound also permits monitoring of IMT over time (useful for evaluating treatment response) and detection of complications such as stenoses and occlusions [219,235].

Integrating ultrasound into fast-track clinics allows diagnosis and treatment initiation within 24 h, significantly reducing the risk of severe ocular complications such as vision loss [219,228,229,240,241,242]. Ultrasound follow-up every six months is recommended for patients with involvement of the upper or lower limb arteries to monitor wall thickness and residual vasculitic activity [243]. A further challenge is differentiating between vasculitis and atherosclerosis: in GCA, wall thickening is concentric, homogeneous, and regular, whereas in atherosclerosis it is often eccentric, irregular, and associated with calcified plaques [244,245,246,247]. Recent studies have demonstrated that the halo sign may occasionally also be present in atherosclerosis, leading to possible false positives [245,246]. Therefore, careful morphological analysis (examining thickening patterns and lesion distribution) and consideration of the clinical context are essential to avoid diagnostic errors [248].

In summary, temporal artery ultrasound is a first-line, non-invasive, and rapid modality with high diagnostic accuracy for GCA and is useful in TAK for monitoring disease activity and vascular complications. Proper implementation requires adequate operator training (at least 30–50 examinations on healthy subjects and five cases of active GCA before routine clinical use) [218,220]. The ability to interpret characteristic sonographic signs (halo sign, compression sign, stenoses, occlusions, macaroni sign), along with the assessment of extra-temporal arteries and the use of CEUS, makes this modality a cornerstone in the modern management of large vessel vasculitis. Finally, complementary techniques such as PET/CT, which assess FDG uptake in vessel walls, can be helpful for comprehensive disease evaluation, although they do not replace ultrasound [249].

## 7. Vertebral Artery Disease

The vertebral artery is classically divided into four segments: V1 (the origin and pre-transverse segment), V2 (the inter-transverse segment), V3 (the vertebral loop preceding its entry into the skull), and V4 (the intracranial segment) [250]. A study of the V1 segment is crucial for identifying the site most affected by stenotic disease. Conversely, evaluating the V2 segment alone is not sufficient to indicate the presence of an ostial stenotic pathology. Alterations in flow velocity and spectral characteristics are observed only at the V2 segment in cases of severe stenosis or occlusion of the vessel’s origin; therefore, other stenoses may not be suspected or diagnosed. The V3 segment, located below the Tillaux space at the retromastoid level, can be easily compressed, resulting in increased and decreased resistances at the V1 segment. This helps precisely position the Doppler sample volume within the vessel, enabling vessel recognition and preventing the common error of detecting flow signals at the thyrocervicoscapular trunk level [250]. Vertebral arteries often exhibit a size discrepancy, with the left vertebral artery being larger than the right in two-thirds of cases [251]. Currently, there are no validated velocity criteria for vertebral arteries, unlike those established for the carotid arteries. However, the literature suggests normal velocity ranges for the V2 segment are between 20 and 60 cm/s. For the V1 segment, an average velocity of 64 cm/s is reported, with a range of 30 to 100 cm/s [252].

### 7.1. Hypoplasia

Among morphological changes, vertebral artery hypoplasia is the most common condition in the general population, with an estimated prevalence of about 11% [253]. Vertebral hypoplasia is defined as a reduction in the vertebral artery diameter to less than 2.5 mm ± 0.4 mm or a difference compared to the contralateral artery with a diameter ratio of 1:1.7 [254]. Vertebral artery hypoplasia poses a risk factor for posterior circulation infarctions, particularly in males and individuals over 65 years of age. Some studies link vertebral artery hypoplasia with increased involvement and extent of stenotic lesions in the posterior circulation [255]. Vertebral artery hypoplasia, defined as a diameter less than 2.5 mm, is regarded as a significant independent risk factor associated with stenoses/occlusions and high-resistance flows (resistance index ≥0.9), regardless of the presence of an accompanying stenosis.

### 7.2. Atherosclerosis

Vertebral artery atherosclerosis is a condition characterised by the accumulation of cholesterol plaques on the arterial walls, leading to reduced blood flow to the brain. This can result in stroke and other neurological complications. The prevalence of vertebral artery atherosclerosis is estimated to be 2–5% in the general population, with a higher incidence in elderly individuals and those with risk factors such as hypertension, diabetes, and smoking.

Doppler ultrasound evaluation of the vertebral artery presents diagnostic challenges. It exhibits a sensitivity of 56.2% but a high specificity of 94.6% for detecting vertebrobasilar stenoses or occlusions. Stenosis identification traditionally relies on the presence of high-resistance flows and a PSV greater than 137.5 cm/s.

Recent studies have refined these diagnostic criteria by measuring both PSV and EDV. Optimal cutoff values for <50%, 50–69%, and 70–99% stenoses have been defined using receiver operating characteristic (ROC) curves. The ratio of intravertebral stenosis PSV to intravertebral distal PSV is the most accurate hemodynamic parameter for assessing stenosis [256] (Table 6).

In conclusion, when used with standardised protocols and combined with PSV/EDV ratio analysis, colour Doppler ultrasound effectively detects intervertebral stenosis.

### 7.3. Uncoarthrosis

Cervical uncoarthrosis, characterised by the formation of osteophytes that compress the vertebral arteries in the V2 segment, can significantly alter vertebrobasilar haemodynamics. Colour Doppler ultrasound may reveal changes such as a reduction in PSV and an increase in the resistive index (RI), indicative of stenosis or compression. While these alterations are useful in diagnosis, they must be interpreted within the patient’s clinical context, integrating ultrasound with imaging studies like MRI and CT for accurate assessment. The detection of such changes is crucial in identifying patients at risk of vertebrobasilar insufficiency, guiding therapeutic decisions. However, it is important to emphasise that, at present, there is a paucity of scientific literature on this topic; therefore, future research is needed to standardise US findings [257].

### 7.4. Vertebral Artery Blood Flow During Cervical Spine Rotation

Doppler ultrasound assessment of the vertebral arteries in individuals presenting with symptoms of vertebrobasilar insufficiency often shows significant hemodynamic changes depending on head position. Specifically, lateral hyperextension of the neck causes a notable decrease in blood flow within the ipsilateral vertebral artery compared to the supine position, while contralateral hyperextension does not produce a significant difference. In people without a history of vascular conditions, these hemodynamic changes seem to be less evident. Additionally, cervical spine rotation results in reduced blood flow in the intracranial vertebral artery, with a more substantial decrease observed on the contralateral side. This effect is more prominent in male subjects than in females, who generally have higher baseline blood flow values. These combined findings suggest that vertebrobasilar hemodynamic changes may play a role in the development of vestibular disorders, especially in patients with undiagnosed positional vertigo [258,259].

### 7.5. Vertebral Artery Dissection

Vertebral artery dissection (VAD) is a major cause of ischemic stroke, particularly in young adults. The combined incidence of vertebral and carotid artery dissections is about 2.6 per 100,000 people, with vertebral artery dissections being three to five times less common than carotid artery dissections [260]. Clinically, patients usually present with neck or occipital pain, severe headache, and sometimes stroke-related symptoms such as unilateral weakness or speech difficulties. However, some may show atypical symptoms or be asymptomatic [261]. Diagnosing VAD can be difficult because of the variable symptoms. While Doppler ultrasound is a useful initial, non-invasive test to evaluate blood flow in the vertebral artery, it often fails to detect important signs like intramural haematoma or false lumen. Therefore, more sensitive imaging techniques, such as MR imaging with fat-saturation sequences or cerebral angiography, are recommended when clinical suspicion is high [262].

## 8. Subclavian Artery Disease

Clinically, a hemodynamic stenosis or occlusion of the subclavian artery is characterised by upper limb fatigue with exercise. The severity of this symptom varies depending on collateral compensation and can progress to difficulties even with minimal effort, such as ‘writer’s cramp.’ If the stenosis is prevertebral, it may also be linked with neurological symptoms, a condition known as “subclavian steal syndrome”. On the right side, a prevertebral obstruction can similarly affect the brachiocephalic artery or common trunk, although this is less common. Physical examination may reveal hypoplasia or absence of pulses in the axillary, brachial, radial, and ulnar arteries. The most easily measured instrumental finding is a systolic pressure difference between the two limbs of more than 20 mmHg [263].

Stenotic or occlusive pathology of the subclavian artery in its prevertebral segment can cause changes in the flow signal direction in the ipsilateral vertebral artery. The steal phenomenon is directly related to the hemodynamic balance between the metabolic demands of the limb and the compensatory capacity of the contralateral vertebral artery. There are conditions where prevertebral stenosis of the subclavian artery is moderate and shows only slight changes in flow signal, which, in the context of altered hemodynamic balance, is referred to as “latent steal.” Conversely, in the “intermittent subclavian steal,” the vertebral flow is antegrade at rest but becomes retrograde during arm stress. In such cases, provocative manoeuvres (arm exercise, reactive hyperaemia) can reveal intermittent steal by increasing ipsilateral arm blood demand. The post-ischemic hyperemia test begins with the patient supine. After recording baseline flow in the ipsilateral vertebral artery via DUS, a pneumatic cuff on the affected arm is inflated to >30–40 mmHg above systolic pressure for 3–5 min to induce ischemia. Upon sudden cuff release, the ischemic arm undergoes reactive hyperemia, marked by a sharp decrease in resistance and an increase in blood flow demand. The duplex probe remains on the vertebral artery, capturing the post-deflation waveform. While normal flow remains stable and antegrade, patients with latent subclavian steal syndrome will show a transient flow reversal in the vertebral artery. The flow can become a to-and-fro pattern or entirely retrograde for several cycles. The waveform typically returns to normal antegrade flow as hyperemia subsides within 15–30 s. Vertebrobasilar symptoms may be provoked during this brief period [264].

In cases of prevertebral occlusive pathology of the subclavian artery, the flow direction in the vertebral artery is reversed, a condition known as “continuous or permanent subclavian artery steal.”

In summary, each type of subclavian steal, when studied with post-ischemic activation of the upper limb, tends to worsen the hemodynamic effect of the “steal” [265,266]. A particularly unique condition that can cause steal affecting the vertebral artery in the absence of prevertebral subclavian pathology is the presence of an iatrogenic arteriovenous fistula (AVF) in dialysis patients. In these cases, the contralateral vertebral artery to the AVF can therefore be involved in all the types of steals described above [265,266].

## 9. Thoracic Outlet Syndrome

Among various pathologies affecting the upper limbs, the most common in young people is upper thoracic outlet syndrome (TOS), caused by compression by structures within an anatomical area traversed by the brachial plexus, the subclavian artery, and the subclavian vein. Neurovascular compression may occur in one of these distinct spaces: (1) the interscalene triangle, formed by the anterior scalene muscle, the middle scalene muscle, and the first rib; (2) the costoclavicular space, bounded by the clavicle, the first rib, and the aponeurosis of the subclavius muscle; (3) the subpectoral space, bounded by the tendon of the pectoralis minor muscle, the coracoid process, and the thorax. TOS can result from anomalous first ribs, abnormal fibrous bands, hypertrophy of scalene muscles, or cervical ribs. Compression can lead to a post-stenotic subclavian artery aneurysm or stenosis/occlusion of the subclavian artery [267]. TOS can be classified into three types based on the affected venous, arterial, or nervous structures: (1) neurogenic TOS, (2) venous TOS, and (3) arterial TOS [268].

Arterial TOS symptoms include digital ischaemia, claudication, pallor, coldness, paraesthesia, and hand pain, but rarely shoulder or neck pain. These symptoms result from arterial emboli, either from mural thrombus in a subclavian artery aneurysm or thrombus distal to subclavian artery stenosis [269]. Compression can also involve the subclavian vein, increasing venous obstruction and/or thrombosis risk. Symptoms may also relate to nerve bundle compression, including pain, weakness, and muscle atrophy [268].

The initial approach to TOS involves clinical assessment through physical examination and provocative manoeuvres. Three commonly used physical exam manoeuvres to diagnose TOS include the Adson test, the elevated arm stress test, the upper limb tension test, the Halstead manoeuvre, the Eden manoeuvre, and the Wright manoeuvre [270]:

- Adson test: the patient is seated upright; the affected shoulder is abducted to 30° with full extension of the elbow. The patient extends their neck while turning their head towards the ipsilateral shoulder and inhaling deeply. In the modified Adson test, the affected shoulder is abducted to 90°. The test is positive if there is a diminished or absent radial pulse. It has a high rate of false positives in diagnosis, even though it is more specific for scalene syndrome or to identify a cervical rib impaction [271,272,273].
- Elevated Arm Stress Test: the patient is seated upright; the arms are abducted to 90°, externally rotated, with elbows flexed to 90°, resembling a “surrender position”. The patient maintains this position while slowly opening and closing their hands for 3 min. The test is positive if there is pain, paraesthesia, numbness, weakness, heaviness, skin colour change, or fatigue in the upper limbs.
- Upper limb tension test: in a supine position, the arms are abducted to 90° with straight elbows; then, the wrist is dorsiflexed, and the head is tilted to each side. The test is positive if neurological symptoms are present.
- Halstead manoeuvre: the patient is seated upright. The examiner palpates the radial pulse and pulls downwards on the limb to be examined while the patient hyperextends and rotates their head away from the side being tested. The test is positive if the radial pulse disappears.
- Eden manoeuvre (or Costoclavicular Maneuver): the patient is seated upright and is instructed to pull the shoulders down and back while slightly extending the neck and chest (military posture). Deep inhalation can help increase compression. The test is positive if there is a reduction or disappearance of the radial pulse.
- Wright manoeuvre: the patient is seated upright. The examiner hyperabducts the arms with external rotation to 90°, maintaining the head straight. The elbow is flexed no more than 45°. The limb is held in this position for about 1 min. The patient can take a deep breath to further provoke symptoms. The test is positive if there is a reduction or disappearance of the radial pulse and/or the onset of paresthesias.

After the clinical assessment, instrumental imaging with US is performed. Cervical spine X-rays are also indicated to exclude supernumerary ribs and thoracic outlet osteoarticular anomalies. MRI and CTA are appropriate second-level investigations, though limited by contraindications and costs. Angiography is indicated only for arterial or venous pathology patients, or when non-invasive diagnostics are insufficient for surgical candidates [274].

### Procedure for the Study of the Thoracic Outlet Syndrome with Ultrasound

Several studies have confirmed the role of US in diagnosing arterial (subclavian) and venous (subclavian and axillary) TOS, while also emphasising the importance of performing the manoeuvres [271,272,273]. The patient is initially assessed in the supine position. The ultrasound examination should include the assessment of the subclavian and axillary artery and vein. Then, the patient is evaluated in a sitting position while performing the provocative manoeuvres. The ultrasound probe is placed longitudinally under the clavicle to examine the subclavian vein. Similarly, the axillary vein is scanned, and colour Doppler and PW Doppler are sampled during the manoeuvres. The absence of flow, an increase in vessel diameter, or the appearance of spontaneous echo contrast are indicative of TOS. Typically, arterial flow is assessed at the level of the axillary artery both at rest and during provocative manoeuvres. Significant compression of the upstream subclavian artery results in reduced flow and changes in the Doppler PW wave morphology. In cases of more severe compression, the flow may disappear entirely.

## 10. Summary

The current document details the operative procedures of SIDV for assessing extracranial artery disease. These recommendations were developed through a narrative review of the literature and a multidisciplinary consensus process to ensure high-quality, safe, and standardised practice in vascular diagnostic laboratories. The document focuses on non-invasive diagnostics, with ultrasound as the primary tool for evaluating carotid, vertebral, subclavian, and innominate arteries.

From a technical perspective, the operative procedures recommend a multiparametric ultrasound approach:

- B-mode imaging is utilised for assessing vessel morphology, intima-media thickness, and plaque.
- Colour and Power Doppler are employed to evaluate flow direction and turbulence.
- Spectral Doppler is used for stenosis quantification, using validated velocity thresholds.

The document also highlights advanced modalities:

- CEUS and MVFI are recognised for their ability to improve plaque characterisation, evaluate intraplaque neovascularisation, and refine cerebrovascular risk stratification.
- 3D US is a tool for characterising plaque morphology and measuring lesions in all planes.

Unfortunately, these methodologies have not yet achieved widespread adoption, as the integration of these innovative approaches into practical applications is hampered by significant barriers, including cost, infrastructure, and technical maturity.

Particular emphasis is placed on moving beyond the degree of stenosis to consider plaque vulnerability features such as echogenicity, surface irregularities, and ulceration, which are more closely linked to clinical risk. The document also includes protocols for post-endarterectomy and post-stenting surveillance, with specific Doppler criteria for restenosis. For vertebral and subclavian arteries, attention is given to flow direction changes and the use of provocation manoeuvres, such as the post-ischemic hyperemia test, to detect intermittent steal.

Finally, the document underscores the importance of appropriateness, patient safety, and operator expertise. The procedures are presented not as legally binding, but as a firm ethical and professional reference for clinicians, to enhance diagnostic accuracy, reproducibility, and the overall quality of care in extracranial vascular imaging.

## Figures and Tables

**Table 1:** The relationship between ECST and NASCET % stenosis.

ICA Stenosis (%NASCET)	ICA Stenosis (%ECST)
30	60
50	70
60	75
70	80
80	90

ICA: Internal Carotid Artery; NASCET: North American Symptomatic Carotid Endarterectomy Trial; ECST: European Carotid Surgery Trial.

**Table 2:** Multiparametric assessment of carotid stenosis (Adapted and modified from Grant EG et al. [146] and Oates CP et al. [143]).

Percentage of Stenosis (NASCET)	PSV ICA (cm/s)	EDV ICA (cm/s)	PSV ICA/PSV CCA Ratio	PSV ICA/EDV CCA (St Mary’s Ratio)
<50	<125	<40	<2	<8
50–59	>125	-	2–4	8–10
60–69	-	-	-	11–13
70–79	>230	>100	>4	14–21
80–89	-	-	-	22–29
>90%	>400	-	> 5	>30
Near-occlusion	High, low-string flow	-	Variable	Variable
Occlusion	No flow	-	Not applicable	Not applicable

NASCET: North American Symptomatic Carotid Endarterectomy Trial; PSV: peak systolic velocity; ICA: internal carotid artery; EDV: end-diastolic velocity; CCA: common carotid artery.

**Table 3:** Recommended duplex ultrasound follow-up schedule after CAS or CEA.

Timing	Follow-Up
48 h	Optional technical check
30 days	Baseline post-operative duplex ultrasound
Every 6 months (first 2 years)	Close surveillance during the period of higher restenosis risk
Annually	Long-term follow up in stable patient at low risk

**Table 4:** Duplex ultrasound velocity criteria for carotid restenosis after CAS and CEA.

Condition	Restenosis	ICA/CCA PSV Ratio	PSV (cm/s)
Post-CAS	>50%	>2.5	>220
Post-CAS	>70%	>3.8	>300
Post-CEA	>50%	>2.25	>213
Post-CEA	>70%	>3.35	>274

ICA: Internal carotid artery; CCA: common carotid artery; PSV: peak systolic velocity; CAS: Carotid Artery Stenting; CEA: Carotid endoarterectomy.

**Table 5:** Ultrasound parameters GCA-TAK.

Ultrasound Parameter	Cut-Off	Specificity	Sensitivity	References
Temporal Artery IMT	Commonly > 0.4 mm Typically 0.5–0.8 mm	≈83~100% if bilateral	≈75% (biopsy-positive) 20% in perivascular/adventitial involvement	[212,214,215,216,217]
Axillary artery IMT	≥1.0 mm (suspicious) ≥1.5 mm (highly suggestive)	High	N/A	[215,226]
Subclavian artery IMT	>1.0 mm	High	N/A	[215,224]
Compression sign	Lack of collapse under transducer pressure	High	Very high (~100%)	[229]
Bilateral halo sign	Present in both frontal and parietal branches	≈100%	≈75%	[213,214]
Macaroni sign (TAK)	IMT > 1.0 mm, homogeneous concentric thickening	High	N/A	[216]

IMT: Intima Media Thickness; TAK: Takayasu Arteritis.

**Table 6:** Duplex ultrasound parameter of vertebral stenosis.

Stenosis Percentage	US Parameter
<50%	PSV (iv-S) ≥ 81.5 cm/s EDV (iv-S) ≥ 24.5 cm/s PSV (iv-S)/PSV (iv-D) ≥ 1.49
50–69%	PSV (iv-S) ≥ 137.5 cm/s EDV (iv-S) ≥ 36.5 cm/s PSV (iv-S)/PSV (iv-D) ≥ 3.14
70–99%	PSV (iv-S) ≥ 216 cm/s EDV (iv-S) ≥ 55 cm/s PSV (iv-S)/PSV (iv-D) ≥ 4.31

PSV: peak systolic velocity; iv-S: intravertebral stenosis; EDV: end diastolic velocity; iv-D: segment distal to the stenosis.

## Data Availability

The raw data supporting the conclusions of this article will be made available by the authors on request.

## Abbreviations

The following abbreviations are used in this manuscript:

2D	Two-Dimensional
3D	Three-Dimensional
ACT	Acceleration time
AVF	Arteriovenous Fistula
B-mode	Brightness Mode
CAD	Carotid Artery Disease
CAS	Carotid Artery Stenting
CCA	Common Carotid Artery
CDI	Colour Doppler Imaging
CEA	Carotid endarterectomy
C-FMD	Cerebrovascular Fibromuscular Dysplasia
CT	Computer Tomography
CTA	Computer Tomography Angiography
CVD	Cardiovascular Disease
CaW	Carotid Web
CEUS	Contrast Enhanced Ultrasound
DEGUM	Deutsche Gesellschaft fur Ultraschall in der Medizin
DSA	Digital Subtraction Angiography
DUS	Duplex Ultrasonography
ECA	External Carotid Artery
ECST	European Carotid Surgery Trial
EDV	End Diastolic Velocity
ESC	European Society of Cardiology
EVAR	Endovascular Aneurysm Repair
FMD	Fibromuscolar Dysplasia
GCA	Giant Cell Arteritis
GSM	Gray-scale Median
Hz	Hertz
ICA	Internal Carotid Artery
IMT	Intima Media Tickness
LVV	Large Vessel vasculitis
KHz	Kilo Hertz
MHz	Mega Hertz
MRA	Magnetic Resonance Angiography
MRI	Magnetic Resonance Imaging
MVFI	Microvascular flow imaging
NASCET	North American Symptomatic Carotid Endarterectomy Trial
PDI	Power Doppler Imaging
PET	Positron Emission Tomography
PRF	Pulse Repetition Frequency
PRP	Pulse Repetition Period
PSV	Peak Systolic Velocity
PWD	Pulsed Wave Doppler
RI	Resistive Index
ROC	Receiver Operating Characteristic
SIDV	Italian Society for Vascular Investigation
STA	Superficial Temporal Artery
TAK	Takayasu Arteritis
TFB	Thin Fluttering Band
TIA	Transient Ischemic Attack
TOS	Thoracic Outlet Syndrome
US	Ultrasound
VAD	Vertebral artery dissection
WHO	World Health Organization
